# An *ARHGAP25* variant links aberrant Rac1 function to early‐onset skeletal fragility

**DOI:** 10.1002/jbm4.10509

**Published:** 2021-06-07

**Authors:** Riikka E. Mäkitie, Petra Henning, Yaming Jiu, Anders Kämpe, Konstantin Kogan, Alice Costantini, Ville‐Valtteri Välimäki, Carolina Medina‐Gomez, Minna Pekkinen, Isidro B. Salusky, Camilla Schalin‐Jäntti, Maria K. Haanpää, Fernando Rivadeneira, John H. Duncan Bassett, Graham R. Williams, Ulf H. Lerner, Renata C. Pereira, Pekka Lappalainen, Outi Mäkitie

**Affiliations:** ^1^ Folkhälsan Institute of Genetics Helsinki Finland; ^2^ Research Program for Clinical and Molecular Metabolism, Faculty of Medicine University of Helsinki Helsinki Finland; ^3^ Molecular Endocrinology Laboratory, Department of Metabolism, Digestion and Reproduction Imperial College London London UK; ^4^ Department of Internal Medicine and Clinical Nutrition Centre for Bone and Arthritis Research, Institute of Medicine, Sahlgrenska Academy, University of Gothenburg Gothenburg Sweden; ^5^ HiLIFE Institute of Biotechnology University of Helsinki Helsinki Finland; ^6^ The Center for Microbes, Development and Health, Key Laboratory of Molecular Virology and Immunology, Institut Pasteur of Shanghai Chinese Academy of Sciences Shanghai China; ^7^ University of Chinese Academy of Sciences Beijing China; ^8^ Department of Molecular Medicine and Surgery and Center for Molecular Medicine Karolinska Institutet Stockholm Sweden; ^9^ Department of Orthopaedics and Traumatology Helsinki University Central Hospital and Helsinki University, Jorvi Hospital Espoo Finland; ^10^ Department of Internal Medicine Erasmus MC, University Medical Center Rotterdam Rotterdam The Netherlands; ^11^ Department of Pediatrics David Geffen School of Medicine at UCLA Los Angeles California USA; ^12^ Endocrinology, Abdominal Center University of Helsinki and Helsinki University Hospital Helsinki Finland; ^13^ Department of Genomics and Clinical Genetics Turku University Hospital Turku Finland; ^14^ Children's Hospital University and Helsinki University Hospital Helsinki Finland

**Keywords:** OSTEOGENESIS IMPERFECTA, OSTEOPOROSIS, BONE MODELING AND REMODELING, CELL/TISSUE SIGNALING ‐ OTHER, GENETIC RESEARCH

## Abstract

Ras homologous guanosine triphosphatases (RhoGTPases) control several cellular functions, including cytoskeletal actin remodeling and cell migration. Their activities are downregulated by GTPase‐activating proteins (GAPs). Although RhoGTPases are implicated in bone remodeling and osteoclast and osteoblast function, their significance in human bone health and disease remains elusive. Here, we report defective RhoGTPase regulation as a cause of severe, early‐onset, autosomal‐dominant skeletal fragility in a three‐generation Finnish family. Affected individuals (*n* = 13) presented with multiple low‐energy peripheral and vertebral fractures despite normal bone mineral density (BMD). Bone histomorphometry suggested reduced bone volume, low surface area covered by osteoblasts and osteoclasts, and low bone turnover. Exome sequencing identified a novel heterozygous missense variant c.652G>A (p.G218R) in *ARHGAP25*, encoding a GAP for Rho‐family GTPase Rac1. Variants in the *ARHGAP25* 5′ untranslated region (UTR) also associated with BMD and fracture risk in the general population, across multiple genomewide association study (GWAS) meta‐analyses (lead variant rs10048745). *ARHGAP25* messenger RNA (mRNA) was expressed in macrophage colony‐stimulating factor (M‐CSF)–stimulated human monocytes and mouse osteoblasts, indicating a possible role for ARHGAP25 in osteoclast and osteoblast differentiation and activity. Studies on subject‐derived osteoclasts from peripheral blood mononuclear cells did not reveal robust defects in mature osteoclast formation or resorptive activity. However, analysis of osteosarcoma cells overexpressing the ARHGAP25 G218R‐mutant, combined with structural modeling, confirmed that the mutant protein had decreased GAP‐activity against Rac1, resulting in elevated Rac1 activity, increased cell spreading, and membrane ruffling. Our findings indicate that mutated ARHGAP25 causes aberrant Rac1 function and consequently abnormal bone metabolism, highlighting the importance of RhoGAP signaling in bone metabolism in familial forms of skeletal fragility and in the general population, and expanding our understanding of the molecular pathways underlying skeletal fragility. © 2021 The Authors. *JBMR Plus* published by Wiley Periodicals LLC on behalf of American Society for Bone and Mineral Research.

## 1 INTRODUCTION

Childhood or adolescence onset osteoporosis is most commonly associated with osteogenesis imperfecta (OI) and qualitative or quantitative defects in type I collagen.^(^ ^1^, ^2^ ^)^ Discovery of novel rare forms of primary osteoporosis and other skeletal disorders have expanded our understanding of the molecular mechanisms governing bone health.^(^ ^1^, ^3^, ^4^ ^)^ Recent genetic findings have shown that defects in pathways regulating bone cell function and extracellular matrix apart from type I collagen can also have detrimental effects on bone quality: defective WNT signaling leads to impaired osteogenesis; mutations in *PLS3*, encoding Plastin 3, affect osteocyte actin cytoskeleton in X‐linked osteoporosis; and defective xylosyltransferase (XYLT2) function leads to severe spinal osteoporosis due to abnormal glycosaminoglycan metabolism.^(^ ^5^, ^6^, ^7^, ^8^ ^)^ Genomewide association studies (GWASs) in large population‐based cohorts have further identified hundreds of genetic loci, containing genes annotated to several bone‐active pathways such as WNT, Osteoprotegerin (OPG)–receptor activator of nuclear factor κB (RANK)–RANK ligand (RANKL), and mesenchymal cell differentiation, as determinants of bone mineral density (BMD), osteoporosis, and fracture risk.^(^ ^4^, ^9^, ^10^, ^11^, ^12^ ^)^ Despite these discoveries the genetic causes of early‐onset osteoporosis still remain inadequately understood.

Here, we present a large three‐generation Finnish family with a severe, early‐onset autosomal dominant inherited skeletal fragility. Affected individuals exhibit multiple low‐energy peripheral and vertebral compression fractures, loss of adult height but normal BMD. After careful clinical and radiographic phenotyping, and exclusion of pathogenic variants in OI‐related candidate genes, we used whole‐exome sequencing (WES) in six family members to identify the causative genetic defect in the family. We identified a novel heterozygous missense variant c.652G>A (p.G218R) in the gene encoding Ras homologous guanosine triphosphatase (RhoGTPase)‐activating protein 25 (*ARHGAP25*, canonical transcript, isoform A, NM_001007231.2, ENSG00000163219, NP_001007232.2), which wholly segregated with the phenotype of skeletal fragility. Functional validation indicated that the variant impairs RhoGTPase‐related cellular functions and GWAS denoted that *ARHGAP25* is significantly associated with BMD in the general population.

## 2 SUBJECTS AND METHODS

### 2.1 Subjects

The index subject was evaluated at age 53 years for recurrent fractures and a positive family history of increased fragility fractures and an OI‐like skeletal disease. As part of our ongoing research program on genetic determinants of early‐onset osteoporosis we recruited the family, both affected and unaffected family members, into a study aiming to identify the genetic cause of their disease. All subjects or their guardian gave signed informed consent before participation in the study. The study protocol, including clinical and genetic studies, was approved by the ethics committee of the Helsinki University Hospital.

### 2.2 Clinical studies, biochemistry and radiological evaluations

We collected medical histories from prior hospital records and by subject interview for fracture history, other skeletal and nonskeletal morbidities, growth and development, past surgeries, and long‐term medications. As part of routine clinical assessment, exclusion of any underlying secondary causes of skeletal fragility (e.g., endocrinological or hematological illnesses) was performed prior to proceeding with genetic analyses. In addition, altogether nine family members underwent a more thorough clinical evaluation for skeletal and extraskeletal characteristics, including biochemistry, BMD, and imaging studies during a study visit at Helsinki University Hospital.

Biochemical parameters were evaluated from peripheral blood and urine in the morning between 8:00 a.m. and 10:00 a.m., after an overnight fast and using second morning void urine for urine samples. Measurements were done at HUSLAB laboratories (Helsinki, Finland). We assessed peripheral blood biochemistry for complete blood count, electrolytes, creatinine, and concentrations of calcium and phosphate, 25‐hydroxyvitamin D (S‐25‐OH‐D; assessed by chemiluminescent immunoassay [CLIA]; Abbott, Deerfield, IL, USA), and parathyroid hormone (PTH; CLIA assay on the IDS‐iSYS fully automated immunoassay system; Immunodiagnostic Systems, Ltd., Bolton, UK). We also measured urinary concentrations of calcium, phosphate, and creatinine. Measured bone turnover markers included serum total alkaline phosphatase (ALP), serum N‐terminal propeptide of type I procollagen (P1NP, marker of bone formation; CLIA, IDS‐iSYS), and urinary N‐telopeptide of type I collagen (U‐NTx, marker of bone resorption; enzyme‐linked immunosorbent assay [ELISA]; Abbott). We also measured serum intact and C‐terminal fibroblast growth factor 23 (FGF23) by a manual enzyme‐linked immunosorbent assay (Kainos Laboratories, Tokyo, Japan) and by ELISA (Biomedica, Vienna, Austria), respectively, and used the manufacturers' reference ranges. For the other analyses, we used previously published corresponding measurements from 35 healthy Finnish children and adults.^(^ ^13^ ^)^

We evaluated BMD with dual‐energy x‐ray absorptiometry (DXA) for lumbar spine (L_1_–L_4_, LS‐BMD), total hip (FN‐BMD), and total body (TB‐BMD) (Hologic Inc., Bedford, MA, USA). All measurements were converted to *T*‐scores and *Z*‐scores. Plain skeletal radiographs were obtained of thoracic and lumbar spine, long bones, and left hand. Vertebral morphology was analyzed from spinal radiographs and compression fractures were graded according to Genant et al.^(^ ^14^ ^)^; a ≥20% decrease in vertebral height was regarded as indicative of a compression fracture. Previously obtained radiological evaluations were reviewed.

### 2.3 Bone histomorphometry

We obtained transiliac bone biopsies of the anterior superior iliac crest from two subjects (II‐2 and II‐4) with a manual drill, trephine inner diameter of 7.5 mm (Rochester Bone Biopsy, Medical Innovations International, Rochester, MN, USA).^(^ ^15^ ^)^ Local anesthesia and intravenous (iv) sedation were used. Both subjects received tetracycline labeling following a standard 2‐10‐2–day scheme before biopsy: administration of 500 mg oral tetracycline three times a day on 2 consecutive days and repeated on 2 more days after a 10‐day interval, 2 days before biopsy.^(^ ^15^ ^)^ Subjects refrained from dairy products, calcium supplements and antacids during labeling to ensure adequate tetracycline absorption.

The bone biopsy samples were collected in 70% ethanol, dehydrated, and then embedded in polymethylmethalcrylate (PMMA) using standard procedures. Static histomorphometric parameters were evaluated in one entire undecalcified 5‐μm section stained with Toluidine blue. Tetracycline labeling was assessed over the entire area of one 10‐μm unstained section. Primary bone histomorphometric parameters were evaluated in trabecular bone under 200× magnification using the Osteomeasure system (Osteometrics Inc., Atlanta, GA, USA). Mineralized bone was defined by dark blue staining areas; pale‐blue seams at least 1.5 μm in width were included in measurements of osteoid. Derived indices were calculated according to standard formulas.^(^ ^16^ ^)^ We used previously reported age‐specific reference values for each parameter to calculate *Z*‐scores.^(^ ^17^ ^)^ Nomenclature and abbreviations follow the recommendations of the American Society for Bone and Mineral Research.^(^ ^16^ ^)^ All histomorphometric analyses were performed by Renata C. Pereira at the David Geffen School of Medicine at the University of California, Los Angeles (UCLA).

### 2.4 Genetic studies

**FIGURE 1:**
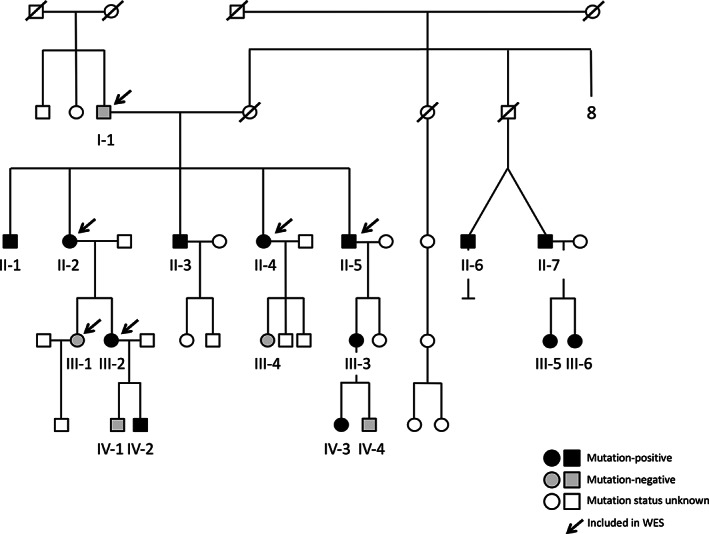
Pedigree of the family with a heterozygous p.G218R *ARHGAP25* mutation. Squares represent males, circles females, black symbols mutation‐positive family members, white symbols unaffected family members, and slashes deceased family members. Subjects included in this study are indicated with codes. All genetically tested family members are indicated with an asterisk. Subjects included in the WES analysis are marked with an arrow. The pedigree has been altered to ensure anonymity. Abbreviation: WES, whole‐exome sequencing.

Prior to our genetic study on the family, the index patient and her immediate family were clinically assessed at Turku University Hospital, Finland. As part of their initial evaluation, known genes for autosomal dominant OI (*COL1A1*, *COL1A2*, and *IFITM5*) were first screened at the Connective Tissue Gene Tests laboratory (CTGT, Allentown, PA, USA) by conventional Sanger sequencing and multiplex ligation‐dependent probe amplification (MLPA) assay; no disease‐causing variants were detected. Subsequently, and after the family was referred for further genetic studies, we selected six family members from three generations including four affected subjects and two healthy and asymptomatic subjects (Figure 1) for WES analysis. Exome sequencing was performed at Oxford Gene Technology (OGT, Oxfordshire, UK) according to their standard methods (www.ogt.com; Supplementary Materials).

We next used the GEMINI (GEnome MINIng) framework (0.19.0)^(^ ^18^ ^)^ for variant exploration. Before undertaking an exomewide search for causative variants in this family, we first evaluated all variants found in the 20 previously reported genes known to underlie primary osteoporosis.^(^ ^19^, ^20^ ^)^ We then selected candidate variants with the following criteria: (i) heterozygous variants present in clinically affected subjects (AII‐2, AII‐4, AII‐5, AIII‐2) and absent in clinically healthy subjects (AI‐1, AIII‐1); (ii) functional variants affecting coding regions or splice junctions; and (iii) an allele frequency of <0.1% in the 125,748 exome sequences and 87,410 whole‐genome sequences (in total from v2 and v3) in the Genome Aggregation Database (gnomAD) database (http://gnomad.broadinstitute.org), the 1000 Genomes Project,^(^ ^21^ ^)^ and the 10,000 Finnish exomes in the Sequencing Initiative Suomi database (SISu; http://www.sisuproject.fi). Using this allele frequency cutoff of 0.1% has good support from the analysis performed by Lek et al.,^(^ ^22^ ^)^ especially when considering a dominant disease. Acknowledging the variability in disease severity within the family, possible compound heterozygous variants were also considered. In silico predictions of the damaging capacity for missense variants were performed using Sorting Intolerant From Tolerant (SIFT),^(^ ^23^ ^)^ Polymorphism Phenotyping version 2 (PolyPhen2; http://genetics.bwh.harvard.edu/pph2/), UMD‐predictor (http://www.umd-predictor.eu/), MutationTaster2,^(^ ^24^ ^)^ Mendelian Clinically Applicable Pathogenicity (M‐CAP) (http://bejerano.stanford.edu/mcap/), Rare Exome Variant Ensemble Learner (REVEL) score,^(^ ^25^ ^)^ and Combined Annotation Dependent Depletion (CADD) scores (https://cadd.gs.washington.edu/). Possible effects of amino acid changes on protein conformation were evaluated using the HOPE web server (https://www3.cmbi.umcn.nl/hope/). The selected candidate variants were confirmed and their segregation in other family members assessed by Sanger sequencing. Primers and protocols are available upon request from the authors.

### 2.5 Array comparative genomic hybridization

Copy number variants (CNVs) were tested with a customized 2 × 400K array (Agilent Technologies, Santa Clara, CA, USA) with genomewide coverage and enriched probes in >300 genes linked to skeletal disease, as described.^(^ ^26^ ^)^ On average, the array covers targeted gene areas with one probe per 100 base pairs (bp) in coding regions and one per 500 in introns and untranslated regions (UTRs). We performed the tests with standard procedures and analyzed results with Agilent Genomic Workbench 7.0 (Agilent Technologies).

### 2.6 GWASs

We interrogated publicly available results from the largest fracture and BMD (DXA and heel ultrasound–estimated) GWAS meta‐analyses performed to screen for associations mapping to the *ARHGAP25* locus (http://www.gefos.org/). One fracture and four BMD site–specific studies were surveyed: any type of fracture (*n* = 264,267; 37,778 cases),^(^ ^27^ ^)^ ultrasound‐derived BMD (eBMD) investigated in the UKBB (*n* = 426,824),^(^ ^11^ ^)^ whole‐body (WB) BMD comprising approximately 66,000 individuals,^(^ ^28^ ^)^ and lumbar spine (LS) and femoral neck (FN) BMD each comprising approximately 33,000 individuals in their discovery phase.^(^ ^29^ ^)^

### 2.7 Tissue and bone cell expression of *Arhgap25*

To analyze expression of *ARHGAP25* in different tissues, we measured *Arhgap25* mRNA levels by quantitative polymerase chain reaction (PCR), detecting both isoforms A and C, in 18 different tissues of male mice (Supplementary Materials). To determine which bone cells express *Arhgap25*, we also measured mRNA levels in cultured murine osteoclasts and osteoblasts; bone marrow macrophages were cultured in M‐CSF alone or with M‐CSF and RANKL to induce osteoclast differentiation. Osteoclastic differentiation was verified by the strong increase in expression of tartrate‐resistant acid phosphatase *Acp5*.

### 2.8 Osteoclast studies from patient‐derived cells

More extensive descriptions of the specific methods are provided in the Supplementary Materials. We obtained peripheral lithium‐heparin blood from three subjects (II‐2, II‐3, II‐4) and three healthy, unrelated, sex‐ and age‐matched controls (C1–C3). CD14^+^ monocytes were isolated from peripheral blood and seeded on 96‐well plates in complete α modified essential medium (α‐MEM) with M‐CSF and RANKL to induce osteoclastogenesis. Osteoclast studies were performed in both plastic wells and on discs of bovine bone to study resorption. Media were replenished every third day and cells were fixed and stained for tartrate resistant acid phosphatase (TRAP) at the indicated time points. Media were saved for analysis of TRAP5b and C‐terminal telopeptides of type I collagen (CTX) (IDS Immunodiagnostic Systems, Boldon, UK). TRAP5b and CTX were analyzed in media collected on day 8, corresponding to the amounts released from days 6 to 8. Resorption pits were visualized by reflective light microscopy following Toluidine blue staining. Actin ring formation was studied after 8 days of culture on bone discs using fluorescence microscopy. Gene expression was analyzed in cells from healthy blood donors, cultured as described above for other cells. *ARHGAP25* and *ACP5* gene expressions were analyzed using TaqMan Assays (Hs01121033_m1 and Hs00356261_m1, Life Technologies, Carlsbad, CA, USA) and the StepOnePlus Real‐Time PCR system (Applied Biosystems, Foster City, CA, USA). *Arhgap25* mRNA expression was also analyzed in in vitro differentiated osteoclasts from mouse bone marrow macrophages using predesigned TaqMan Assay Mm00615449_m1 with 18S ribosomal RNA as an internal control.

### 2.9 Protein studies

More extensive descriptions of the specific methods are provided in the Supplementary Materials. In brief, we tested the effect of the *ARHGAP25* mutation on protein function in terms of actin organization, cell spreading, and activity of ARHGAP25‐targeted Rac1. For this, we used *ARHGAP25* c.631G>A p.G211R mutant, which corresponds to the G218R variant identified in the patients but expressed in isoform C rather than isoform A of *ARHGAP25* (NM_001166276.2, NP_001159748.1). These two splice variants differ only at their first coding exon (i.e., the N‐termini): isoform C is seven amino acids shorter than isoform A as a sequence of 15 amino acids at the N‐terminus is replaced by a different sequence of eight amino acids. The critical domain regions, including the GTPase‐activating protein (GAP) domain harboring the mutated residue, are identical in the two isoforms and the functional activities of the two mutants are therefore considered the same (Supplementary Materials). For clarity, we have used the p.G218R nomenclature throughout the text and results.

As a positive control, we used an isoform C green fluorescent protein (GFP)‐ARHGAP25 p.R193A construct, with a previously described inactive GAP function and defective Rac1 activity.^(^ ^30^ ^)^ Human osteosarcoma cells were maintained and cultured under standard conditions and transfected with the different plasmids. Rac1 activity was measured with a Rac1 gold‐labeled immunosorbent assay Small GTPase Activation Assays (G‐LISA) absorbance‐based biochemical assay kit (Cytoskeleton, Denver, CO, USA) and the absorbance values compared between the control cells and cells overexpressing wild‐type ARHGAP25, G211R‐ARHGAP25 (corresponding to the patient G218R mutant) or R193A‐ARHGAP25 mutants as described.^(^ ^31^ ^)^ To examine the effects on actin cytoskeleton, immunofluorescence microscopy was applied to visualize F‐actin in the cells using Alexa Fluor 568‐phalloidin (Thermo Fisher, Waltham, MA, USA), and the cells were imaged using wide‐field fluorescence microscopy (DM6000B; Leica, Espoo, Finland). To test cell spreading, we cultured cells on cover slips (either 1 h on fibronectin‐coated or 5 h on noncoated) and measured cell areas. Structural modeling of the location of the G218R mutation within ARHGAP25 was performed using Phyre2 server (http://www.sbg.bio.ic.ac.uk/phyre2/html/page.cgi?id=index).

### 2.10 Statistical analyses

For the cell spreading assay, statistical analyses were performed with SigmaPlot 11.0 (Systat Software, Berkshire, UK). For the osteoclast assays, statistical comparisons were made by Student's *t* test between M‐CSF and M‐CSF+RANKL–treated cells and between subjects and their respective age‐ and sex‐matched controls. Association between the variant and the family's skeletal phenotype was statistically evaluated using linkage analysis and statistical logarithm (LOD) score calculation (see Methods in Supplementary Materials). Difference was considered statistically significant with *p* value <0.05.

## 3 RESULTS

### 3.1 Clinical phenotype

**FIGURE 2:**
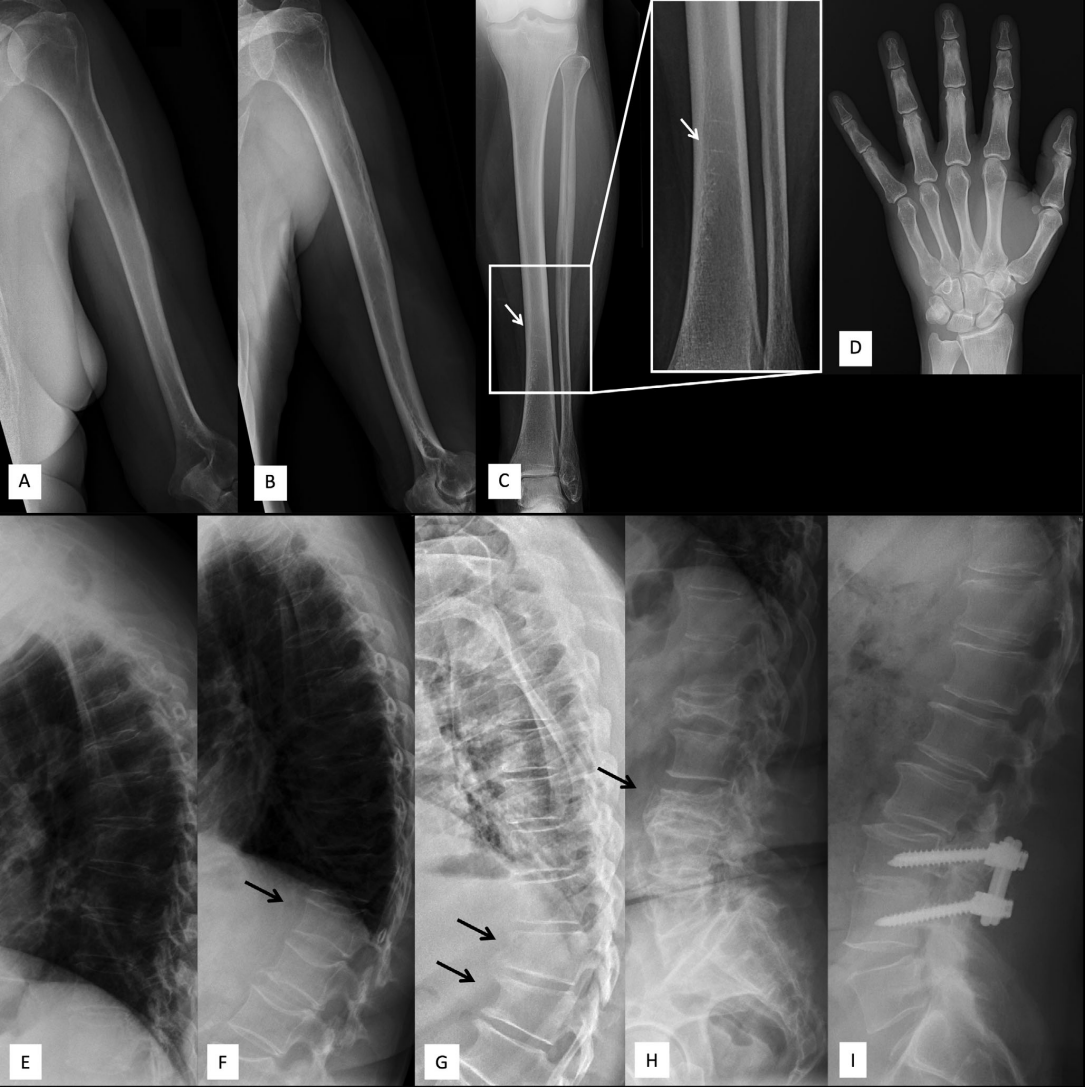
Long bone, hand, and spinal radiographs of four subjects with a heterozygous p.G218R *ARHGAP25* mutation. Images show visible trabeculation and horizontal growth arrest–lines (white arrow) indicating poor mineral content and disturbed bone turnover, and multiple vertebral compression fractures (black arrows), particularly near the thoracolumbar junction of the spine, and subsequent exaggerated thoracic kyphosis and straightened lumbar lordosis. Upper extremity images of (*A*) 69‐year‐old female (II‐2, index) and (*B*) 64‐year‐old female (II‐4). Lower extremity image (*C*) and left hand (*D*) of 69‐year‐old female. Thoracic spine radiographs of (*E*) 69‐year‐old female, (*F*) 64‐year‐old female, and (*G*) 61‐year‐old male (II‐6). Lumbar spine radiographs of (*H*) 69‐year‐old female (II‐2, index), and (*I*) 61‐year‐old male.

Initially, the family comprised five siblings who were all considered affected by skeletal fragility with varying phenotypes (Figure 1, Table 1). The index subject (II‐2), a currently 69‐year‐old female, had a history of over 60 low‐energy fractures beginning at the age of 8 years. Peripheral fractures included >10 ribs, femur, tibia, more than two humeri, radius, sternum, clavicle, metacarpals, metatarsals, and multiple compression fractures. These most often resulted from low‐energy trauma such as coughing or bending over; no high‐energy traumas were known. She had debilitating back pain, kyphotic stature, and a 10‐cm loss of adult height (Figure 2, Table 1). She had joint pain and increased joint laxity and slightly blue sclerae. Dental records stated dental fragility with recurrent tooth chipping and caries, periodontitis, and horizontal bone loss. Prior treatment with bisphosphonates resulted in no improvement in skeletal health because her BMD remained static and she suffered new fractures throughout treatment. Furthermore, fracture healing appeared delayed; following her clavicle fracture, evidence of callus formation was not seen until 4 months after the initial diagnosis. She also presented with psoriasis and dermatitis herpetiformis, but there was no history of diabetes or long‐term glucocorticoid treatment.

**TABLE 1:** Clinical and bone densitometry findings in 13 mutation‐positive and five mutation‐negative family members with heterozygous missense mutation p.G218R in *ARHGAP25*

Code	Sex	Age	BMD *Z*‐score			Fractures		Adult height loss (cm)	Back pain	Joint pain	Osteoporosis medication	Autoimmune/autoinflammatory diseases
			LS	FN	WB	Peripheral	Vertebral					
Mutation‐positive subjects (*n* = 13)												
II‐2^a^	F	69	3.7	−0.3	0.4	>60	4	10	Y	Y	Y	Psoriasis, dermatitis herpetiformis
II‐1	M	70	−0.3	−0.2	N/A	3	0	N/A	Y	Y	N	Psoriasis
II‐3^a^	M	67	1.5	−0.5	−0.3	8	2	6	Y	Y	N	Unspecific abdominal pain
II‐4^a^	F	64	0.2	1.1	0.0	4–5	5	3	Y	Y	Y	Psoriasis, celiac disease, Crohn's disease
II‐5	M	61	0.0	−1.3	N/A	>6	0	0	Y	Y	Y	Hypothyroidism, rheumatoid arthritis
III‐2	F	42	+0.9	0.0	N/A	3	0	0	Y	Y	N	Hypothyroidism
IV‐2	M	7	N/A	N/A	N/A	0	0	0	N	N	N	Hypothyroidism
III‐3^a^	F	40	−0.7	−0.3	0.2	1	2^c^	0	N/A	N/A	N	–
IV‐3^a^	F	11	0.0	0.0^d^	0.4	0	0	0	N	N/A	N	–
II‐7^a^	M	62	−0.8^b^	0.3	−0.6	0	5	0	Y	Y	N	Unspecific abdominal pain
II‐6^a^	M	62	1.0	0.7	0.2	3	4	0	Y	Y	N	Unspecific abdominal pain
III‐5^a^	F	36	−0.3	−0.9	0.4	0	0	0	Y	Y	N	Unspecific abdominal pain
III‐6^a^	F	33	−0.3	−0.4	0.0	0	0	0	Y	Y	N	–
Mutation‐negative subjects (*n* = 5)												
I‐1	M	93	−1.0	−2.8	N/A	0	1	0	N	N	N	–
III‐1	F	45	+0.7	−0.6	N/A	1	0	0	N	N	N	–
IV‐4^a^	M	8	0.9	3.2^d^	1.2	0	0	0	N/A	N/A	N	Unspecific abdominal pain
IV‐1	M	9	N/A	N/A	N/A	0	0	0	N	Y	N	–
III‐4	F	40	−0.8	−0.5	N/A	1	0	0	N	Y	N	Rheumatic arthritis

Abbreviations: F, female; FN, femoral neck; LS, lumbar spine; M, male; N, no; N/A, not available; WB, whole body; Y, yes.

^a^ Clinically evaluated during a study visit at the Helsinki University Hospital.

^b^ Only for L_1_ and L_2_.

^c^ Decrease in vertebral height <20%.

^d^ Measurement for total femoral head.

Her four siblings also had multiple fractures of both large and small long bones and vertebrae following low‐energy traumas, slightly blue sclerae, and similar dental problems (Table 1). All had normal hearing, growth, and pubertal development, normal intellect, and no apparent facial dysmorphias or skeletal malformations. Many of them similarly presented with various autoimmune or autoinflammatory disorders. None had diabetes or received long‐term glucocorticoid treatment.

Biochemistry showed normal blood count, including normal leucocyte and platelet counts, although two subjects had increased hemoglobin and four others had hemoglobin concentrations close to the upper limit. Plasma calcium and serum 25‐OH‐D concentrations were normal (Table 2). Six out of nine had mild hypophosphatemia whereas only two had markedly increased PTH; the other biochemical values in these two individuals were normal (Table 2). Serum intact and C‐terminal FGF23 were normal in four subjects in which they were measured (Supplementary Figure S1). Bone turnover markers ALP, P1NP, and crosslinked amino terminal telopeptide type I collagen (INTP) were normal in all subjects. None had hypercalciuria or hyperphosphaturia.

**TABLE 2:** Biochemistry findings in 13 mutation‐positive and five mutation‐negative family members with heterozygous missense mutation p.G218R in *ARHGAP25*

Code	Sex	Age	Hb	Ca	Pi	PTH	D25OH	FGF23, i	ALP	P1NP	Cr	U‐INTP	U‐Ca	U‐Pi	Hypercalciuria
Mutation‐positive subjects (*n* = 13)															
II‐2^a^	F	69	152	2.34	**0.67**	40	99	N/A	67	32	74	22	0.99	6.9	N
II‐1	M	70	*168*	N/A	N/A	N/A	N/A	N/A	N/A	N/A	N/A	N/A	N/A	N/A	N/A
II‐3^a^	M	67	161	2.31	**0.76**	37	82	N/A	82	35	87	2.9	2.6	38.6	N
II‐4^a^	F	64	143	2.30	1.03	52	79	N/A	86	58	69	36	7.9	48.0	N
II‐5	M	61	151	1.26	N/A	N/A	125	N/A	N/A	N/A	N/A	N/A	N/A	N/A	N/A
III‐2	F	42	146	N/A	N/A	N/A	N/A	N/A	49	N/A	77	N/A	N/A	N/A	N/A
IV‐2	M	7	148	N/A	N/A	N/A	N/A	N/A	151	N/A	N/A	N/A	N/A	N/A	N/A
III‐3^a^	F	40	145	2.33	1.20	29	96	N/A	28	28	N/A	2.5	N/A	N/A	N
IV‐3^a^	F	11	138	2.35	1.44	34	79	N/A	172	740	N/A	820	N/A	N/A	N
II‐7^a^	M	62	154	2.22	**0.67**	*109*	103	54.05	48	N/A	100	30	5.86	29.7	N
II‐6^a^	M	62	*174*	2.42	**0.56**	51	81	47.92	54	49	95	32	2.61	12.9	N
III‐5^a^	F	36	147	2.19	**0.75**	*94*	62	41.12	84	N/A	59	27	2.09	27.6	N
III‐6^a^	F	33	138	2.24	**0.72**	63	87	34.34	77	N/A	65	35	1.8	13.9	N
Mutation‐negative subjects (*n* = 5)															
I‐1	M	93	144	N/A	N/A	N/A	N/A	N/A	N/A	N/A	N/A	N/A	N/A	N/A	N/A
III‐1	F	45	140	N/A	N/A	N/A	N/A	N/A	N/A	N/A	N/A	N/A	N/A	N/A	N/A
IV‐4^a^	M	8	128	2.36	1.58	32	90	N/A	131	653	N/A	850	N/A	N/A	N
IV‐1	M	9	N/A	N/A	N/A	N/A	N/A	N/A	N/A	N/A	N/A	N/A	N/A	N/A	N/A
III‐4	F	40	134	N/A	N/A	N/A	N/A	N/A	N/A	N/A	N/A	N/A	N/A	N/A	N/A

*Notes*: Supranormal values are in italics and subnormal values are in bold. Normal ranges according to HUSLAB Laboratory (F/M): B‐Hb (g/L): 4–7 years, 112–147; 8–11 years, 116–154; 12–15 years, 120–154/123–170; >18 years, 117–155/134–167; P‐Ca (calcium, mmol/L): 2.15–2.51; P‐Pi (phosphate, mmol/L): 0.76–1.41; fP‐PTH (ng/L): 10–65; D25OH (nmol/L): > 50; ESR (mm/h): <15; FGF23 (intact) according to Immutopics International and Kainos Laboratories: 8.2–54.3 pg/mL. P‐ALP (U/L): 10–11 years, 115–435/115–335; 12–13 years, 90–335/125–405; 16–18 years, 35–125/55–330; >18 years, 35–105; S‐P1NP (intact; bone formation marker, μg/L): 10–11 years, 388–1094/328–1169; 12–13 years, 82–650/194–1146; 16–17 years, 25–148/67–436; >18 years, 17–124/21–110; Cr (μmol/L): 6–12 years, 10–76; 13–16 years; 15–90/20–95; >18 years; 50–90/60–100; U‐INTP (bone resorption marker, nmol/mmol Cr): 1–13 years, 307–1763; 14–17 years, 55–378/102–1048; >18 years, <65 (premonepausal)/<63; postmenopausal women, 21–116^(^ ^66^ ^)^; hypercalciuria (urine calcium creatinine ratio): >0.7.

Abbreviations: ALP, alkaline phosphatase; B‐Hb, hemoglobin; CR, creatinine; D25OH, 25‐hydroxy vitamin D; ESR, erythrocyte sedimentation rate; F, female; FGF23, fibroblast growth factor 23; M, male; P1NP, procollagen I N‐terminal propeptide; PTH, parathyroid hormone; U‐INTP, urine type I collagen cross‐linked N‐telopeptide; Y, yes; N, no; N/A, not available.

^a^ Clinically evaluated during a study visit at the Helsinki University Hospital.

DXA‐derived LS‐BMD, FN‐BMD, and WB‐BMD were normal or slightly elevated in all subjects (Table 1). Skeletal radiographs showed loss of bone mineral with visible trabeculation (Figure 2). Long bones had thin diaphyses with overtubulated metaphyseal ends (Figure 2*A*–*C*). Some bones had horizontal growth arrest–lines as a sign of poor mineral content (Figure 2*D*). Compression fractures were common especially in the lower thoracic and upper lumbar vertebrae (Figure 2*E*–*I*). No skeletal complications specific to OI, such as abnormal angulation of long bones, formation of hyperplastic callus, or atypical soft tissue calcifications were apparent. Joints were normal without deformity. Although fracture healing seemed to be delayed, as mentioned above in the first paragraph, old fractures appeared to have ultimately healed normally.

### 3.2 Bone histomorphometry

**FIGURE 3:**
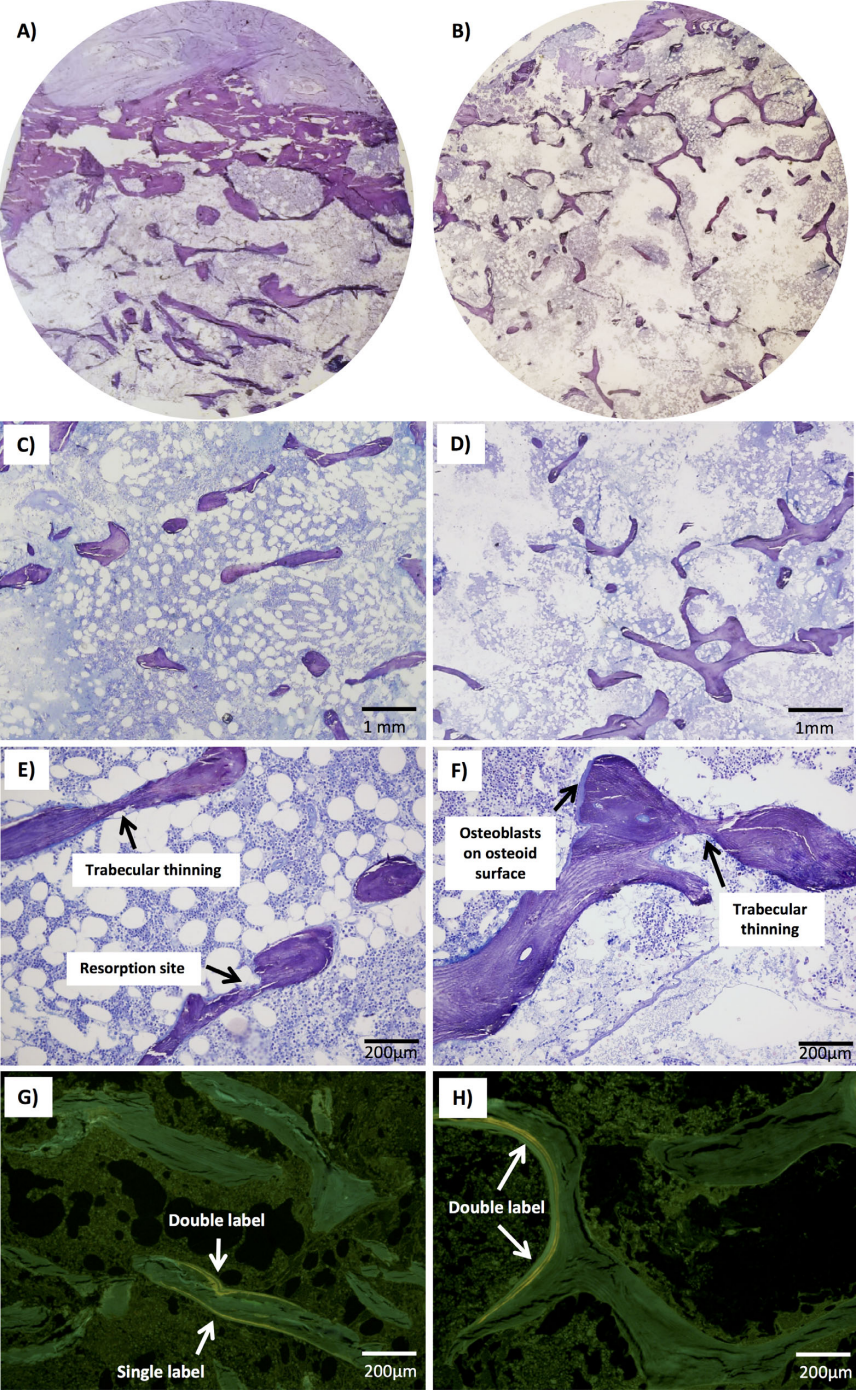
Bone histomorphometric findings in two subjects with a heterozygous mutation p.G218R in *ARHGAP25*. Toluidine blue staining of biopsies from (*A*,*C*,*E*) 69‐year‐old female (II‐2, index) and (*B*,*D*,*F*) 64‐year‐old female (II‐4, sister) showing loss of interconnection among trabecular plates, trabecular thinning and reduction in trabecular number, and reduction in number of resorption sites. Biopsies from both (*G*) II‐2 and (*H*) II‐4 also had reduced tetracycline uptake and only a few visible double labels.

We obtained transiliac bone biopsies and bone histomorphometric data for the index (II‐2; a 69‐year‐old female) and her affected sister (II‐4; a 64‐year‐old female) (Table 3, Figure 3A‐H). Both had previously received bisphosphonates but the medications were discontinued prior to biopsy: the index had received zoledronic acid which had ended 5 years prior to biopsy and the sister had received alendronic acid which had ended 6 years prior to biopsy. The histology and histomorphometric findings were similar in both biopsies. The cancellous bone volume was severely reduced, which was due to the overall reductions in trabecular number and thickness, leading to loss of interconnections between trabeculae. The trabecular bone surface covered with osteoid (unmineralized bone) and the osteoid thickness were decreased. The surface covered by osteoblasts or by osteoclasts were similarly reduced. For patient II‐2, in a few areas with very thin osteoid‐only flat osteoblasts or lining cells were visible and for patient II‐4 a few areas had “plump” osteoblasts on the osteoid surface. Tetracycline uptake was also reduced in both biopsy samples and only a few double labels were visible (Figure 3). Consistent with the other findings indicating low bone turnover, the eroded surface, mineralizing surface, and the mineral apposition rate (calculated from the distance between two double labels) were decreased, indicating, with the rest of the findings, low bone turnover. These findings also excluded osteomalacia. Because cortical bone was only preserved in one of the samples, cortical parameters could not be fully assessed.

**TABLE 3:** Bone histomorphometric findings in two subjects with a heterozygous missense mutation p.G218R in *ARHGAP25* and a pathological fracture history

Parameter	II‐2 (index, 69 years)			II‐4 (sister, 64 years)		
	Value	Reference mean ± SD (F, 65–74 years)	*Z*‐score	Value	Reference mean ± SD (F, 55–64 years)	Z‐score
BV/TV (%)	6.76	19.56 ± 5.62	**−2.28**	8.20	20.79 ± 4.37	**−2.88**
OV/BV (%)	0.14	1.2 ± 0.87	−1.22	0.11	2.17 ± 1.14	−**1.81**
O.Th (μm)	2.43	8.31 ± 1.99	**−2.95**	6.30	9.16 ± 1.94	−1.47
OS/BS (%)	1.78	14 ± 6.64	−**1.84**	7.80	16.7 ± 6.99	−1.27
Ob.S/BS (%)	0.00	3.11 ± 2.75	−1.13	0.24	6.05 ± 3.83	**−1.52**
ES/BS (%)	2.16	3.66 ± 1.69	−0.89	0.93	4.14 ± 2.12	**−1.51**
Oc.S/BS (%)	0.34	0.59 ± 0.73	−0.34	0.15	0.82 ± 0.80	−0.84
Tb.Th (μm)	63.35	131.3 ± 28.10	**−2.42**	73.00	133 ± 34.40	**−1.74**
Tb.Sp (μm)	873.00	690.5 ± 178.00	1.03	822.00	626.9 ± 94.40	*2.07*
Tb.N (n/mm)	1.07	1.49 ± 0.29	−1.45	1.10	1.59 ± 0.23	**−2.13**
MS/BS (%)	0.70	5.79 ± 4.38	−1.16	4.50	7.77 ± 4.20	−0.78
MAR (μm/d)	0.467	0.477 ± 0.078	−0.09	0.471	0.526 ± 0.044	−1.27
BFR/BS (μ^3^m/μ^2^m/year)	1.19	10.1 ± 7.99	−1.12	7.80	15.0 ± 8.0	−0.90
Mlt (days)	13.25	68 ± 55.5	−0.99	23.17	43.5 ± 24.5	−0.83
FB.V (%)	Traces			None		

*Notes*: *Z*‐scores were calculated using age‐specific reference values according to Recker et al.^(^ ^17^ ^)^ Values above 1.5 SD are in italics and values below −1.5 SD are in bold.

Abbreviations: BFR/BS, bone formation rate/bone surface; BV/TV, bone volume/total volume; ES/BS, eroded surface/bone surface; F, female; FB.V, fibrosis; MAR, mineral apposition rate; Mlt, mineralization lag time; MS/BS, mineralizing surface/bone surface; O.Th, osteoid thickness; Ob.S/BS, osteoblast surface/bone surface; Oc.S/BS, osteoclast surface/bone surface; OS/BS, osteoid surface/bone surface; OV/BV, osteoid volume/bone volume; SD, standard deviation; Tb.N, trabecular number; Tb.Sp, trabecular separation; Tb.Th, trabecular thickness.

### 3.3 Genetic results

**FIGURE 4:**
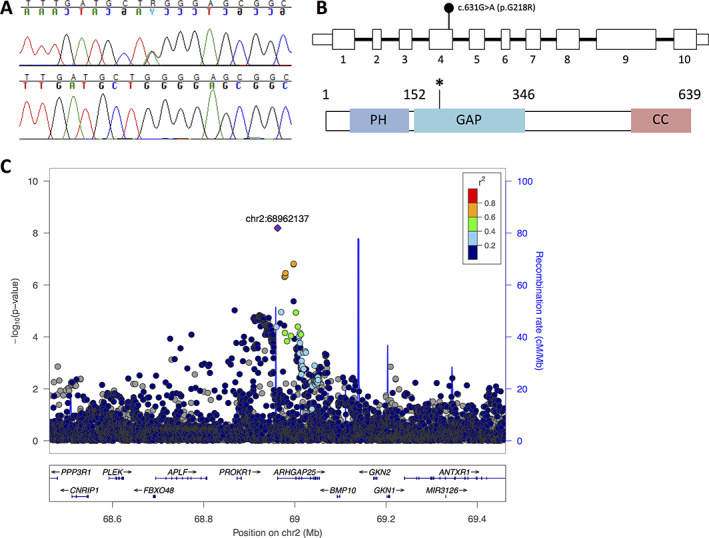
Genetic findings in a family with a heterozygous p.G218R *ARHGAP25* mutation. (*A*) Sequence image of the heterozygous point mutation in the index patient (II‐2) and normal sequence from a healthy family member (I‐1). (*B*) Schematic presentation of *ARHGAP25* and the location of the heterozygous missense mutation c.652G>A (p.G218R) in exon 4. (*C*) Regional TB‐BMD association plot for the *ARHGAP25* locus. Each circle represents one SNP in the locus and its y‐coordinate the significance for the TB‐BMD GWAS meta‐analysis (*n* = 66,628) reported by Medina‐Gomez et al.^(^ ^28^ ^)^ Different colors indicate varying degrees of pairwise linkage disequilibrium with the top marker [rs10048745] according to the 1000 Genomes – CEU population. Abbreviations: CEU, Utah residents (Centre d'Etude du Polymorphisme Humain [CEPH]) with Northern and Western European ancestry; GWAS, genomewide association study; TB‐BMD, total body–bone mineral density.

No pathogenic variants were found in the 21 genes known to cause classical OI or OI‐like primary osteoporosis (Supplementary Table S1). Filtering of the WES data yielded seven variants (Supplementary Table S2) that segregated with the phenotype: six were missense variants and one a frameshift insertion. Of these, we omitted five variants due to their association with known human genetic diseases with very different phenotypes to that of our family. Of the remaining two variants, the missense variant p.L108P in *ZBTB9* was Sanger sequenced from all family members' DNA and identified as a false variant. Thus, we focused on *ARHGAP25* c.652G>A (p.G218R) (Canonical transcript, isoform A, ENSG00000163219, NP_001007232.2, NM_001007231.3) and confirmed its sequence and segregation in the entire family by Sanger sequencing (Figure 4A and B). Overall, we screened 12 additional family members and found eight to harbor the mutation (Figures 1 and 4A and B). For all these eight subjects, the mutation segregated with skeletal fragility.

The identified *ARHGAP25* missense variant p.G218R is novel with no carriers identified in the Exome Aggregation Consortium (ExAC), gnomAD, and SISu databases. It is predicted to be damaging by all five prediction programs: damaging by SIFT (score 0.036), probably damaging by PolyPhen2 (score 0.958), pathogenic by UMD Predictor (pathogenicity 100), disease‐causing by MutationTaster2 (disease‐causing probability 0.9999), and possibly pathogenic by M‐CAP (score 0.100). It also has a CADD score of 27.7 and a REVEL score of 0.551 (specificity 91%). Automated analysis of the mutant protein by HOPE indicated that the mutant residue is larger and more hydrophilic than the wildtype residue and introduces a new positive charge that can repel neighboring residues. The mutant amino acid also lies near a highly conserved region and on the surface of the protein in the Rho‐GAP domain.^(^ ^32^ ^)^ Taken together, these data suggest that the mutation is likely damaging to the protein and may disrupt its interactions with other molecules. Linkage analysis for association between the variant and the family's skeletal phenotype yielded a significant LOD score in three out of four scenarios (LOD max 3.08–4.86 at theta = 0), supporting the variant being disease‐causing (Supplementary Figure S5).

Because of the unexplained finding of a low serum phosphate in several subjects, we further investigated all genes known to be associated with phosphate homeostasis disorders.^(^ ^33^ ^)^ Subsequently, six variants were found in three different genes, but all were located in intronic or 3′UTR regions and none segregated with the phenotype (Supplementary Table S3).

Further, in addition to analysis of the exome sequencing data, we assessed possible structural variants that might have not been identified by WES. We detected no significant gene dosage imbalances with array–comparative genomic hybridization (CGH). Thus, no CNVs were found as the cause of the disease in the family and were omitted as a probable genetic cause.

### 3.4 GWASs

One common variant (rs10048745, minor allele frequency [MAF] = 0.25) showed a genomewide significant (GWS) association with WB‐BMD (*p* = 6.44 × 10^−9^) (Figure 4*C*). Each copy of the minor allele rs10048745‐A was associated with a 0.039 standard deviation (SD) decrease in WB‐BMD. Similarly, consistent associations were observed for the A‐allele in relation to LS‐BMD and FN‐BMD (LS: beta = −0.050 SD, *p* = 1.03 × 10^−6^; FN: beta: −0.036 SD; *p* = 5.21 × 10^−6^). The same rs10048745‐A allele variant in *ARHGAP25* was also associated with heel ultrasound–derived BMD (beta = −0.014, *p* = 4.8 × 10^−12^). Altogether 11 other variants in high LD with the top variant were found associated at GWS level in the UK Biobank (Supplementary Table S4). Finally, the rs10048745 variant was associated at nominal significance level with any type of fracture (odds ratio [OR] = 1.1017; 95% confidence interval [CI], 1.002–1.032; *p* = 0.02).

### 3.5 Tissue and bone cell expression of *Arhgap25*

Analyses of *Arhgap25* mRNA expression in mouse tissues revealed the gene to be highly expressed in cortical bone and vertebral body, with only spleen and thymus of all 18 tissues analyzed having a higher level of expression (Supplementary Figure S2). In cultured murine osteoclasts and osteoblasts *Arhgap25* was more robustly expressed in macrophages and decreased during osteoclast differentiation (Supplementary Figure S3*A*). *Arhgap25* was also expressed in cultured murine calvarial osteoblasts and the expression was strongly increased when cells were incubated in osteogenic medium (Supplementary Figure S3*C*). *Arhgap25* expression was increased 13‐fold from day 2 to day 7, whereas the osteoblastic gene *Alpl* was increased approximately fivefold (Supplementary Figure S3*D*). The relative expression of *Arhgap25* was higher in macrophages and osteoclasts than in osteoblasts at the investigated time points (Supplementary Figure S3*E*).

### 3.6 Osteoclast studies of patient‐derived cells

With the positive finding of high *Arhgap25* mRNA expression in murine osteoclasts, we analyzed *ARHGAP25* mRNA expression in human osteoclast progenitor cells and mature osteoclasts cultured from peripheral blood of healthy blood donors. When osteoclast formation from peripheral blood osteoclast progenitor cells was induced by RANKL, the mRNA expression of ACP5 was, as expected, induced at 72 h when multinucleated TRAP‐positive cells are formed (Supplementary Figure S4*A*). *ARHGAP25* mRNA was expressed in M‐CSF–stimulated human monocytes. *ARHGAP25* mRNA was significantly lower at both 24 and 72 h of culture in RANKL (Supplementary Figure S4*B*), indicating a potentially functional role of ARHGAP25 in osteoclast differentiation or activity. A lower expression of *Arhgap25* mRNA in in vitro differentiated osteoclasts compared to progenitors cultured in M‐CSF was also confirmed in cultures using mouse bone marrow macrophages as osteoclast progenitors (Supplementary Figure S3*A*).

**FIGURE 5:**
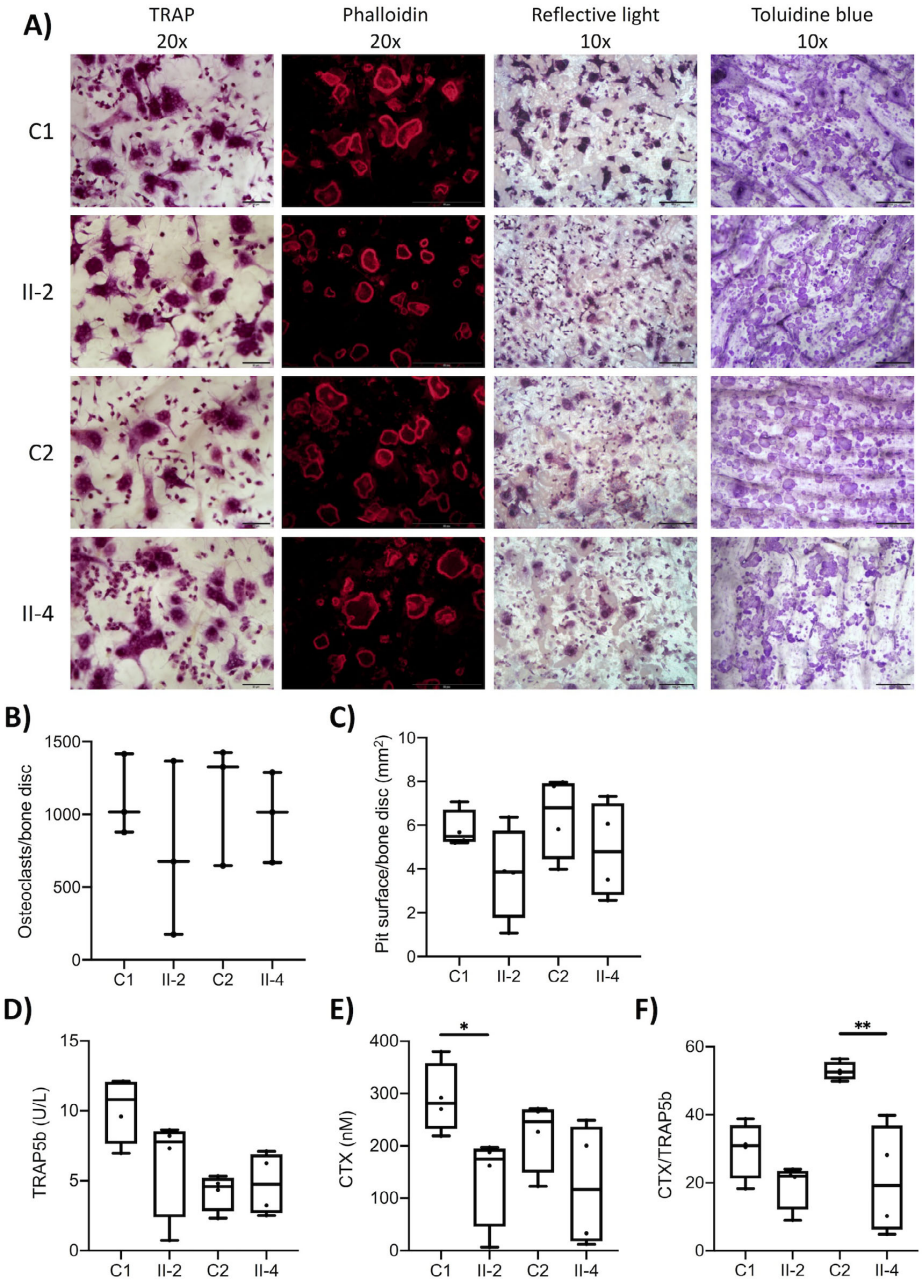
RANKL‐induced osteoclastogenesis, actin ring formation and bone resorption. (*A*) Photographs of TRAP‐ and phallodin‐stained osteoclasts after 8 days of culture in M‐CSF and RANKL on bone discs (TRAP, scale bars = 20 μm; phalloidin, scale bars = 50 μm). Resorption pits visualized by reflective light (resorption pits visible as darker areas) and Toluidine blue staining of bone discs after 8 days of culture in M‐CSF and RANKL (scale bars = 100 μm). (*B*) Number of osteoclasts and (*C*) pit surface per bone disc at day 8 of culture. (*D*) TRAP5b, (*E*) CTX, and (*F*) CTX/TRAP5b ratio in culture media collected between days 6 and 8 of culture in M‐CSF and RANKL. *n* = 3 culture wells/individual in *B* and *n* = 4 culture wells/individuals in *C*–*F*. *p < 0.05, **p < 0.01 (Student's *t* test between patient and respective age‐ and sex‐matched control). Abbreviations: CTX, C‐terminal telopeptides of type I collagen; M‐CSF, macrophage colony‐stimulating factor; RANKL, receptor activator of nuclear factor κB ligand; TRAP, tartrate resistant acid phosphatase.

We then evaluated whether the mutated ARHGAP25 affects osteoclastogenesis in the subjects. We hypothesized that the histological finding of reduced trabecular bone volume could be due to increased osteoclast differentiation or function. We were, however, unable to observe differences in mature osteoclast formation or morphology when RANKL‐stimulated CD14+ monocytes from subjects and healthy controls were cultured on plastic dishes (Supplementary Figure S4*C*). Also, we were unable to observe differences in mature osteoclast formation or morphology between cells from subjects and healthy controls when the cultures were performed on bone discs and stained for TRAP (Figure 5*A*). Similar numbers of osteoclasts were formed (Figure 5*B*), an observation supported by analysis of TRAP5b in the media (Figure 5*D*). Further, we did not observe any difference in actin ring formation in mature osteoclasts between subjects and healthy controls (Figure 5*A*). Osteoclasts from both subjects and controls formed numerous resorption pits as shown by reflective light and Toluidine blue staining (Figure 5*A*). The mutated ARHGAP25 did not affect mature osteoclast activity as assessed by the resorbed surface (Figure 5*C*) and by the amount of CTX released from bone to media (Figure 5*E*). When the CTX/TRAP5b ratio was calculated to assess the resorptive activity per osteoclast, osteoclasts from one healthy control (C2) seemed to have higher resorptive activity but the other healthy control and the subjects had similar CTX/TRAP5b ratio (Figure 5*F*). These results indicate the ARHGAP25 mutation does not increase osteoclastogenesis but may inhibit bone resorption activity slightly. This finding, however, does not adequately account for the increased bone loss observed in the patients.

### 3.7 Protein studies

**FIGURE 6:**
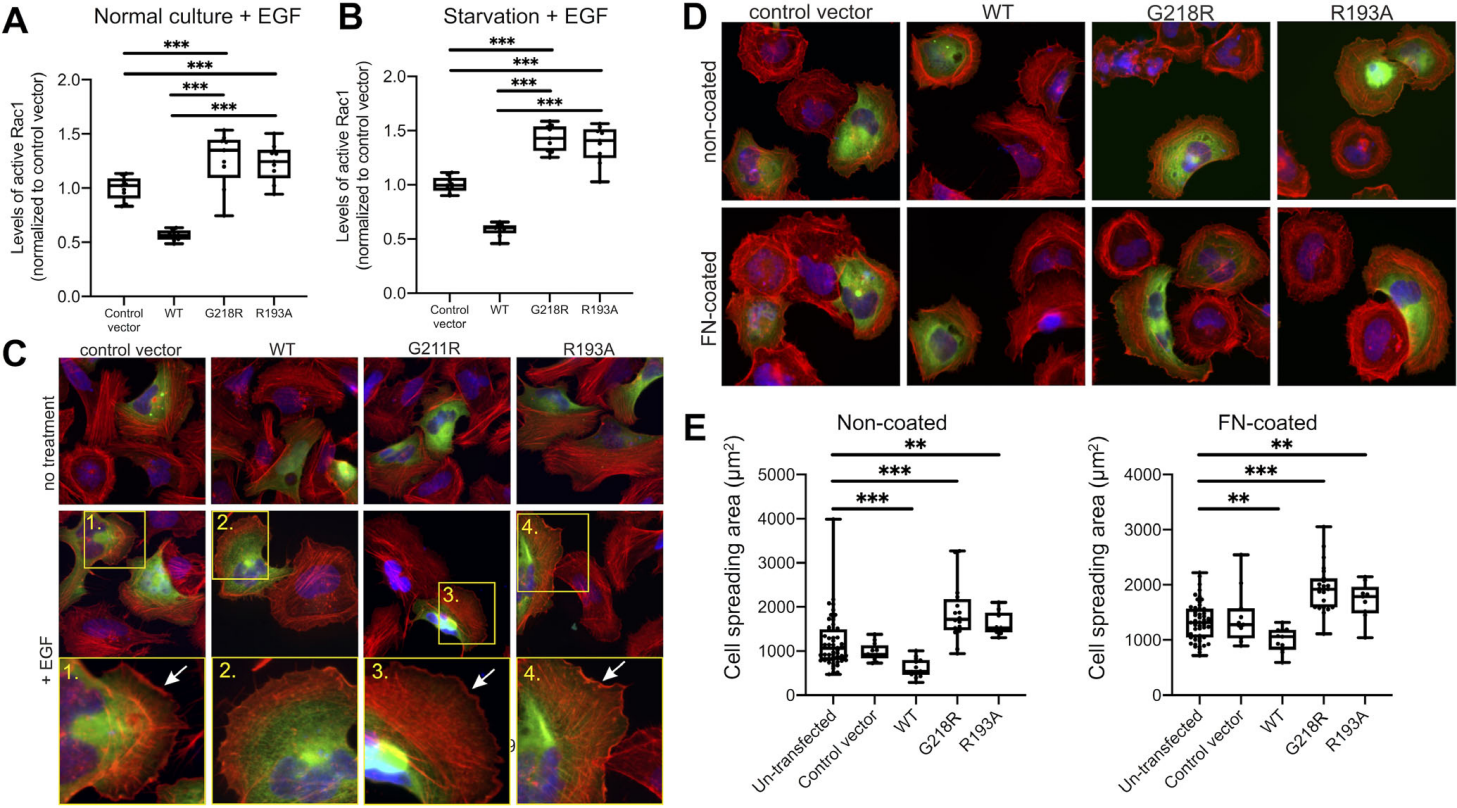
The mutation p.G218R in *ARHGAP25* regulates the GTPase activity of Rac1. G‐LISA analysis of the levels of active Rac1 in U2OS cells transfected with control vector, ARHGAP25 wild‐type, ARHGAP25 G218R, and ARHGAP25 R193A after EGF‐stimulation either in (*A*) normal culture condition or (*B*) after starvation. Data are from three independent experiments and three technical repetitions in case, and the values were normalized to the ones of cells transfected with the control vector. (*C*) Effects of GFP fusions of wild‐type, G218R, and R193A ARHGAP25 on EGF‐induced membrane ruffling. Stimulation was carried out with 0.1 μg/ml EGF for 30 min. Transfected cells can be recognized by expression of green fusions proteins, nuclei were stained with DAPI (blue), and F‐actin was visualized with Alexa‐568–labeled phalloidin (red). Numbered magnified regions (corresponding to the yellow boxes in low magnification images) display examples of EGF‐induced ruffles that are indicated by arrows. (*D*) Immunofluorescence microscopy analysis demonstrating of the effects of wild‐type, G218R, and R193A ARHGAP25 on cell spreading on non‐coated (5‐h incubation, upper panel) and on fibronectin‐coated (1‐h incubation, lower panel) cover slips. (*E*) Quantification of cell spreading area after 5‐h incubation on non‐coated (left panel) and 1‐h incubation on fibronectin‐coated cover slips (right panel) from three independent experiments. **p < 0.01, ***p < 0.001 (Mann‐Whitney‐Wilcoxon rank‐sum test). Abbreviations: DAPI, 4,6‐diamidino‐2‐phenylindole; EGF, enhanced green fluorescent; G‐LISA, gold‐labeled immunosorbent assay; GFP, green fluorescent protein; GTPase, guanosine triphosphatase.

Although *Arhgap25* mRNA expression was also observed in osteoblasts, patient osteoblasts were unfortunately not available for further studies. We therefore performed protein functional studies and cellular assays in osteoblast‐like cells. For this, we expressed the full‐length wild‐type ARHGAP25 and its mutant forms R193A (positive control) and G218R as GFP‐fusion proteins in U2OS osteosarcoma cells to test their GTPase activating effect by G‐LISA assay. As expected, overexpression of wild‐type ARHGAP25 significantly decreased the level of active GTP‐bound Rac1 in U2OS cells after enhanced green fluorescent (EGF) stimulation both in normal culture conditions and following starvation (Figure 6*A*,*B*). Consistent with a previous study,^(^ ^34^ ^)^ the G218R mutation resulted in decreased ARHGAP25 GAP activity against Rac1 and, consequently, an increased level of active Rac1. Importantly, the G218R mutant resulted in comparable increase in the level of active Rac1 (Figure 6*A*,*B*), indicating that the p.G218R mutation efficiently inhibits the GAP activity of ARHGAP25.

The Rho subfamily of small GTPases are central regulators of actin dynamics and organization in eukaryotic cells. Among these, Rac1 has been linked to the assembly of cytoskeletal actin filament networks at the cell periphery to produce lamellipodia and membrane ruffles that enable cell spreading and migration.^(^ ^35^ ^)^ To confirm the GTPase‐activating effect of ARHGAP25 on Rac1 in vivo, U2OS cells were transiently transfected with GFP‐tagged ARHGAP25. Overexpression of ARHGAP25 significantly abolished the EGF‐induced ruffling as visualized by phalloidin staining (Figure 6*C*), whereas robust membrane ruffling was observed in the cells transfected with vectors expressing GAP‐deficient R193A‐mutants or the G218R‐mutants (Figure 6*C*). These mutations also affected cell spreading on both fibronectin‐coated and non‐coated surfaces. Cells transfected with a vector expressing wild‐type ARHGAP25 displayed predominantly a rounded shape and spread slowly, whereas cells expressing the R193A‐mutants and G218R‐mutants spread significantly more rapidly compared to the nontransfected cells (Figure 6*D*,*E*). These results provide evidence that the p.G218R mutation in *ARHGAP25* results in loss of ARGHAP25 GAP activity against Rac1, leading to elevated Rac1 activity and consequently increased membrane ruffling and cell spreading in cells expressing mutant ARHGAP25.

**FIGURE 7:**
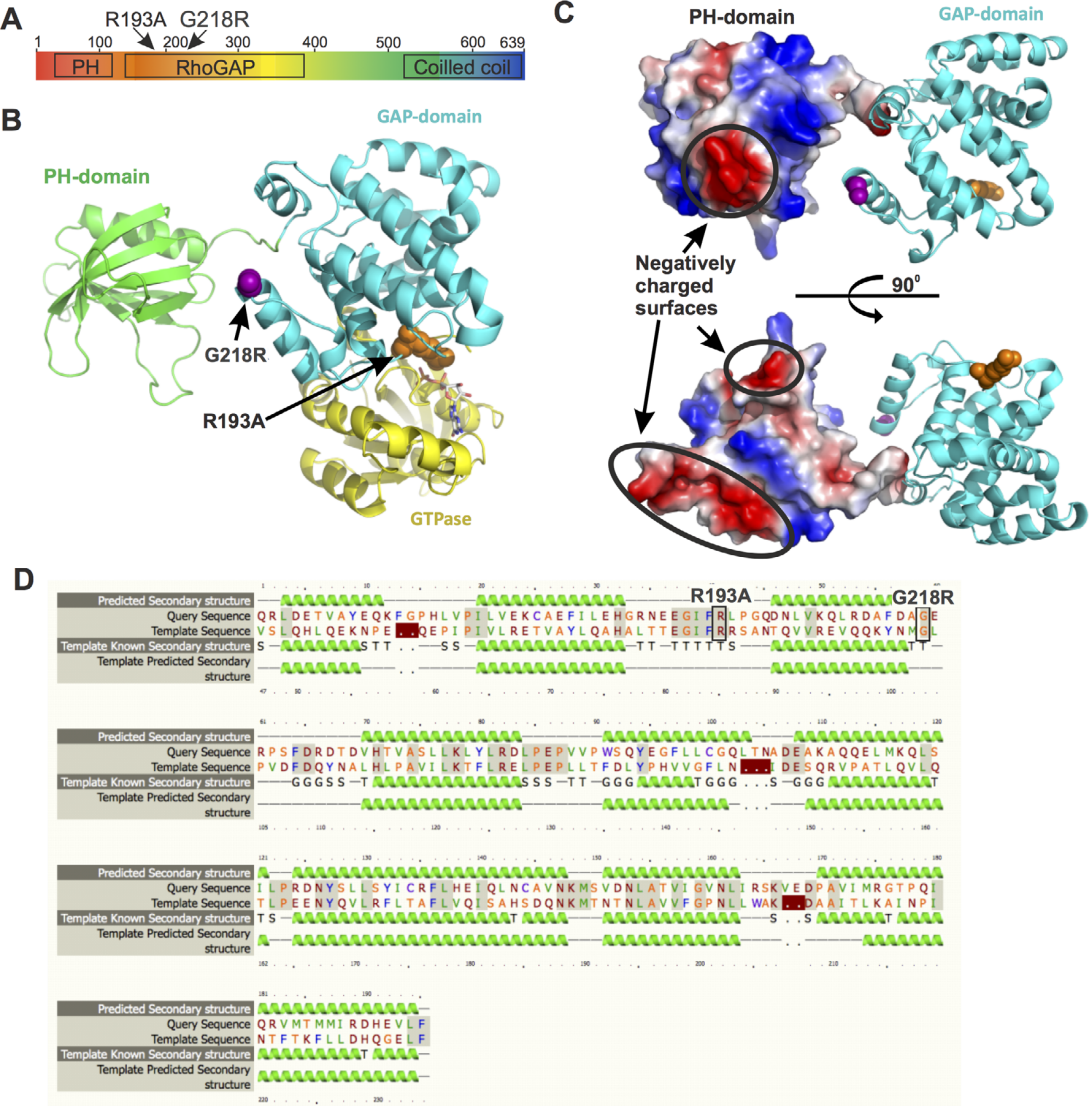
Structural basis of inactivation of *ARHGAP25* by the p.G218R mutation. (*A*) Domain architecture of ARHGAP25. (*B*) Combined model of PH (green) and GAP (cyan) domains of ARHGAP25. The locations of R193 and G211 (corresponding to the G218R mutation in patients) in the structure are indicated by orange and magenta, respectively. The PH domain is separated from the GAP domain by a short (5–7 residues) linker. This allows certain degree of rotation of the domains relative to each other but keeps them still close enough for interaction. (*C*) Surface potential representation of PH domain shows negatively charged areas (red) that may interact with the positively charged arginine in the G218R‐mutant (magenta) and thus keep the ARHGAP25 in an inactive conformation. (*D*) Sequence alignment of the GAP domains of ARHGAP25 (query sequence) and RhoGAP (template sequence, Uniprot: Q07960), used for modeling, shows high sequence similarity and high degree of secondary structure element conservation between two sequences. Abbreviations: GAP, GTPase‐activating protein; GTPase, guanosine triphosphatase; PH, pleckstrin homology; Rho, Ras homologous.

ARHGAP25 consists of an N‐terminal pleckstrin homology (PH) domain followed by a GAP domain and a C‐terminal coiled coil sequence^(^ ^36^ ^)^ (Figure 7*A*). Both R193 and G218R residues are located in the GAP domain of ARHGAP25. By structural modeling (Figure 7*B*,*C*), the previously identified inactivating mutant R193A is located at the interface between the GAP domain and the GTPase. Although the glycine residue mutated in G218R is well‐conserved in ARHGAP25 proteins (Figure 7*D*), the residue is not located at the actual interface between the GAP domain and GTPase (Figure 7*B*). This indicates that a mutation in G218R affects the activity of ARHGAP25 indirectly, most likely by locking the full‐length protein in an inactive conformation through promoting interactions of the GAP domain with adjacent protein domains (Figure 7*C*,*D*). Together, these experiments demonstrate that the *ARHGAP25* G218R mutation results in defects in the protein's function and that the subsequent increase in Rac1 activity affects cell morphology and behavior.

## 4 DISCUSSION

We describe a large Finnish family with multiple family members affected by an early‐onset, autosomal dominant form of bone fragility with markedly increased propensity to fracture. Using WES we identified a novel heterozygous missense variant p.G218R in *ARHGAP25* that segregated with the phenotype. The mutation is predicted pathogenic and disease‐causing, and molecular modeling indicated the mutation lies in close proximity to a critical enzymatic domain and is predicted to interfere with interactions between ARHGAP25 and its molecular partners. A leading *ARHGAP25* variant (rs10048745) is further shown by GWAS in the general population to be associated with BMD (*p* = 6.44 × 10^−9^) and increased risk for any type of fracture (*p* = 0.02). Further experiments revealed high *Arhgap25* mRNA expression in mouse bone tissues, and functional assays indicated a role for ARHGAP25 in bone cell Rac‐dependent cytoskeletal dynamics, membrane ruffling, and cell spreading, although this effect could not be confirmed in subject‐derived peripheral blood cells. To our knowledge, this is the first description of an ARHGAP25‐related bone disorder, demonstrating a novel form of monogenic skeletal disorder resulting from abnormal GTPase activity and giving evidence for a critical role for RhoGAP signaling in bone metabolism and pathology.

RhoGTPases are small signaling G proteins belonging to the Ras superfamily.^(^ ^37^, ^38^, ^39^, ^40^ ^)^ They are further divided into three subgroups: Rho, Rac, and Cdc42. All three subgroups relay information from the extracellular environment to intracellular cascades that regulate actomyosin fibers and focal adhesions (RhoA), assembly of branched cytoskeletal actin filament networks at the cell periphery to create lamellipodia and membrane ruffles (Rac1), and thin actin‐rich plasma membrane protrusions called filopodia (Cdc42).^(^ ^37^, ^38^, ^39^, ^40^ ^)^ ARHGAPs are RhoGTPase‐activating proteins that catalyze the cycle between the active and inactive states of these small intracellular GTPases. In a resting state, the RhoGTPases are bound to cytosolic guanine nucleotide‐dissociation inhibitors (GDIs). Following an extracellular signal, RhoGTPases are released and moved to the plasma membrane, where guanine nucleotide exchange factors (ARHGEFs) activate them to a GTP‐bound form. In their active state, the RhoGTPases go on to regulate key cell functions, such as cell cycle progression, cytoskeletal dynamics, and cell apoptosis. Once the functional goal has been accomplished, ARHGAPs help switch the RhoGTPases back to inactive GDP‐bound state, thus terminating their functional activity.^(^ ^37^, ^38^, ^39^, ^40^ ^)^ RhoGTPases are considered to act as molecular switches and transmit crosstalk between different signaling pathways, such as WNT signaling.

ARHGAP25 is one of the 53 described human RhoGAP domain–containing proteins.^(^ ^37^, ^39^ ^)^ ARHGAP25 belongs to the same family with ARHGAP22 and ARHGAP24 and is similarly considered to act as a specific GAP for Rac1 RhoGTPases.^(^ ^25^, ^35^ ^)^ ARHGAPs, in addition to regulating RhoGTPases, are also suggested to be deployed downstream of key regulatory molecules and serve as signaling intermediates in several other intracellular cascades.^(^ ^41^, ^42^, ^43^ ^)^ This is further supported by the presence of multiple domains and functional motifs in ARHGAP proteins, which may interact with other interplaying factors.^(^ ^41^, ^42^, ^43^ ^)^ ARHGAP22 and ARHGAP24, regulated by Rho‐associated protein kinases (ROCKs), are especially implicated in cancer cells and in the transition of mesenchymal stem cells to amoeboid cells by inhibiting Rac1 activity,^(^ ^44^ ^)^ whereas ARHGAP25 has been shown to regulate phagocytosis in neutrophilic granulocytes.^(^ ^35^ ^)^ However, ARGHAP25, with its ubiquitous expression, as also demonstrated by our results, is likely involved in a variety of biological processes.^(^ ^39^, ^45^ ^)^

Although the role of ARHGAP25 in bone metabolism has not been explored previously, various studies provide evidence for the involvement of RhoGTPases in bone metabolism, especially in osteoclastogenesis, osteoclast morphogenesis, and osteoclast function.^(^ ^46^ ^)^ RhoGTP signaling is important for osteoclast podosome and actin‐ring organization, which is key to osteoclast migration and adherence to specific bone areas, and bone erosion in resorption pits.^(^ ^47^, ^48^, ^49^, ^50^ ^)^ Razzouk et al.^(^ ^51^ ^)^ showed how diminishing Rac1 function in osteoclasts caused a shift to a rounder shape due to distracted actin assembly and decreased resorptive capacity. Several studies have also evaluated the in vivo effects of aberrant RhoGTPase function on bone metabolism^(^ ^46^ ^)^; Croke et al.,^(^ ^52^ ^)^ Ito et al.,^(^ ^53^ ^)^ and Itokowa et al.^(^ ^54^ ^)^ all report that deletion of RhoGTPases in mice causes severe osteopetrosis with increased trabecular number and reduced bone resorption. These changes were specifically due to abnormalities in osteoclast resorption as the number of osteoclasts was normal or only slightly elevated. Furthermore, Wang et al.^(^ ^48^ ^)^ showed that deletion of Rac1 in monocytes/osteoclast progenitors causes a mild osteopetrotic phenotype with increased trabecular bone due to decreased osteoclastogenesis in vitro and in vivo, specifically in RANK‐mediated osteoclast formation. By contrast, several reports show that the importance of Rac proteins in bone metabolism is not limited to the osteoclastic lineage but also extends to the regulation of osteoblast differentiation and cell function.^(^ ^55^, ^56^ ^)^ Accordingly, Huck et al.^(^ ^57^ ^)^ found that deletion of *Rac1*, exclusively at preosteoblastic stage, leads to decreased osteoblast proliferation and increased apoptosis, and in vivo deletion of *Rac1* in mice results in decreased histomorphometric measures of osteoblast function, osteoid thickness, and BMD, which could be mediated through WNT signaling, as demonstrated by Wan et al.^(^ ^58^ ^)^ Because macrophages are an important source of inflammatory cytokines, it is also possible that some of the *ARHGAP25* variant's negative effects on bone homeostasis could be indirect and mediated by abnormal production of such cytokines.^(^ ^59^ ^)^ The skeletal phenotype of the Finnish family reported here could, therefore, result from defects in both osteoclast and osteoblast function.

Although the phenotype and severity of symptoms varied among the affected subjects, most had severe bone fragility with multiple peripheral and vertebral compression fractures despite relatively normal BMD. Similar skeletal fragility in conjunction with normal or even increased BMD has been described in several other instances in OI or monogenic osteoporosis.^(^ ^60^, ^61^ ^)^ Also, similar phenotypic variability has been observed in other forms of monogenic osteoporosis, including osteoporosis related to WNT1.^(^ ^62^ ^)^ This could partly be accounted for by interplaying lifestyle and environmental factors, as well as possible other genetic factors modifying the surfacing phenotype. The bone biopsies had significantly reduced bone volume, trabecular number, trabecular thickness, osteoid surface, and osteoid thickness, as well as decreases in eroded surface or surface covered by osteoblasts or osteoclasts, together implying resembling a low bone turnover status. This most could arise from simultaneously impaired bone formation and resorption. This finding is further supported by the low uptake of tetracycline, suggesting a low state of bone remodeling. The scarcity of active bone cells and reduced bone turnover would also explain the poor response to bisphosphonate treatment, as seen in other low‐turnover forms of primary osteoporosis.^(^ ^1^, ^5^, ^7^ ^)^ Despite the observed severe skeletal abnormalities, metabolic bone markers were normal, as were serum and urinary calcium. By contrast, phosphate was decreased in several affected family members while PTH and FGF23 remained in normal range and bone biopsies excluded osteomalacia. Furthermore, analysis of genes associated with disorders of phosphate homeostasis identified no causative variants. The significance of this and its relation to the underlying skeletal disorder remain to be clarified and warrant further studies.

The identified missense variant is novel, predicted as disease‐causing by several prediction programs, and also segregated wholly in the pedigree. Our linkage analysis also suggests that the variant is responsible for the family's skeletal phenotype. Further support for the role of ARHGAP25 in bone health is given by the finding of a leading variant rs10048745 in the 5′UTR of *ARHGAP25* that has been implicated in BMD variability in the normal population. Although the variant identified in the family does not seem to affect BMD, as implied by the normality in BMD in the patients, the GWAS common variant is located directly upstream of *ARHGAP25* and could therefore have a differential regulatory role. The same variant is also associated with expression of ARHGAP25 in different tissues as evidenced in the expression quantitative trait loci (eQTL) summary reported by Genotype‐Tissue Expression Portal (GTEx 2017; https://gtexportal.org/home/). Although expression data for bone tissue is not available in the surveyed database, our in vitro analyses revealed high *Arhgap25* expression in mouse bone tissues.

Our functional protein assays demonstrated that the missense mutation p.G218R affects the GTPase‐activating ability of the protein, resulting in loss of its ARHGAP25 GAP activity against Rac1 and therefore increased Rac1 activity, even though the mutation does not reside in the active site of the GAP domain but on the protein surface. The mutation also leads to a replacement of a glycine, which is a small amino acid, with a positively charged arginine on an otherwise negatively charged surface of the GAP domain. This may change the protein conformation and abolish some flexibility critical for the protein function. It is therefore likely that, in addition to inhibiting the GAP function, the p.G218R mutation leads to abnormal protein folding and/or disruption of the GAP domain's interactions with neighboring proteins. Such downstream consequences may further be driven by impaired degradation and cytoplasmic accumulation of the mutant protein. The precise molecular mechanisms and the intracellular location and processing of the mutant protein remain to be determined in future studies.

Although *ARHGAP2*5 mRNA was expressed in M‐CSF–stimulated human monocytes, functional experiments with osteoclast progenitor cells from affected subjects and healthy controls showed no difference in osteoclast differentiation or morphology but a tendency to decreased function, an observation which alone cannot explain the decreased bone mass in the patients. The lack of robust differences could be partly due to the small sample size, use of peripheral blood–derived osteoprogenitor cells, and using single blood sample collection timepoint, because these cells might not reflect the exact condition of osteoclast progenitors in the bone marrow. Furthermore, considering the variability in severity of the skeletal manifestations, it could be that the implications for cellular functions are milder and may therefore not be seen as major differences between a small number of subject and control cell lines. The lack of in vitro osteoclast defects might also suggest that the decrease in eroded surface and osteoclast surface in vivo could be due to indirect effects mediated by other cells in the local in vivo environment necessary for initiation of osteoclast formation. It is possible that the local production of osteoclastogenic cytokines such as M‐SCF and RANKL by osteoblasts and/or osteocytes is decreased, which could result in decreased osteoclast formation and resorption. Because we were unable to study patient‐derived osteoblasts, the interaction between bone cells involved in osteoclast formation remains to be investigated. We also showed that *Arhagp25* is expressed in murine periosteal bone cell cultures and that expression is increased during osteoblastic differentiation. The patients had decreased osteoid thickness and surface and decreased osteoblast surface. Hence, it is possible that the mutation affects osteoblast differentiation and function. Furthermore, considering also the normality in measured metabolic bone markers, the skeletal manifestations could also arise from abnormal osteocyte function. While relatively little is known about the importance of RhoGTPases—and even less of ARHGAP25—in osteocytes, their role in organization and dynamics of the actin cytoskeleton in the osteogenic lineage is more established.^(^ ^63^, ^64^ ^)^ This too warrants further investigation. Last, some of the skeletal characteristics could also result from the long‐term consequences of abnormal skeletal development. Suzuki et al.^(^ ^65^ ^)^ have shown that the dose of Rac1 is crucial for normal cartilage development and that excess Rac1 activity results in growth plate disorganization, which would support our finding of increased Rac1 activity and early onset of fractures in our patients.

We recognize the lack of clarity on the molecular mechanisms underlying the family's skeletal pathology and the need for more extensive functional studies to fully elucidate the role of ARHGAP25 in bone metabolism. Our results indicate a potential role for the protein in bone cell function, and although our experiments with peripheral blood–derived bone cells showed no significant difference, analysis with bone marrow cells and with a greater sample size might show the contrary. We were unable to assess the function of patient‐derived osteoblasts, which would have been important given our findings of *Arhgap25* expression in osteoblastic cells. Despite extensive screening of other patients with primary skeletal fragility, we found no other individuals or families with the same gene defect, limiting the number of study subjects, clinical data, and available blood and tissue samples. The low number of available bone biopsies (*n* = 2), destructed cortices in biopsies, lack of age‐ and sex‐matched healthy control samples, and the unblinded nature of analysis also limited thorough analysis of bone tissue changes. However, and despite these limitations, given the rarity of such monogenic forms of bone disorders the current findings provide valuable and novel insight into the molecular mechanisms underlying bone health and the role of ARHGAP signaling in the pathogenesis of skeletal fragility.

In conclusion, we identified a novel heterozygous missense mutation p.G218R in *ARHGAP25* in a large Finnish family with severe, early‐onset, and dominantly inherited bone fragility. The clinical and functional evaluations indicate that the mutation affects bone cell function, most likely resulting in low bone resorption and turnover and subsequently skeletal fragility with an increased propensity to fractures. Further understanding of the consequences of abnormal activity of ARHGAP25 in bone would require expanded studies in additional mutation‐positive individuals and in a murine model of the disease. Such studies have the potential to reveal new insights into the molecular pathways regulating bone health and pathogenesis.

## DISCLOSURES

The authors, Riikka E. Mäkitie, Petra Henning, Yaming Jiu, Anders Kämpe, Konstantin Kogan, Alice Costantini, Ville‐Valtteri Välimäki, Carolina Medina‐Gomez, Minna Pekkinen, Isidro B. Salusky, Camilla Schalin‐Jäntti, Maria K. Haanpää, Fernando Rivadeneira, J. H. Duncan Bassett, Graham R. Williams, Ulf H. Lerner, Renata C. Pereira, Pekka Lappalainen and Outi Mäkitie, declare no conflicts of interest.

## AUTHOR CONTRIBUTIONS

**Riikka E. Mäkitie** study design and conduct, data analysis, writing‐review and editing, **Petra Henning** study design and conduct, data analysis, writing‐review and editing, **Yaming Jiu** study design and conduct, data analysis, writing‐review and editing, **Anders Kämpe** study conduct, data analysis, writing‐review and editing, **Konstantin Kogan** study conduct, data analysis, writing‐review and editing, **Alice Costantini** study conduct, data analysis, writing‐review and editing, **Ville‐Valtteri Välimäki** study conduct, writing‐review and editing, **Carolina Medina‐Gomez** study conduct, data analysis, writing‐review and editing, **Minna Pekkinen** writing‐review and editing, **Isidro B. Salusky** writing‐review and editing, **Camilla Schalin‐Jäntti** writing‐review and editing, **Maria K. Haanpää** writing‐review and editing, **Fernando Rivadeneira** data analysis, writing‐review and editing, **J. H. Duncan Bassett** data analysis, writing‐review and editing, **Graham R. Williams** data analysis, writing‐review and editing, **Ulf H. Lerner** study design and conduct, data analysis, writing‐review and editing, **Renata C. Pereira** data analysis, writing‐review and editing, **Pekka Lappalainen** study design and conduct, data analysis, writing‐review and editing, **Outi Mäkitie** study design and conduct, data analysis, writing‐review and editing.

5

### PEER REVIEW

The peer review history for this article is available at https://publons.com/publon/10.1002/jbm4.10509.

## Supporting information


**Appendix S1**: Supplementary Materials

## References

[1] Mäkitie RE , Kämpe AJ , Taylan F , Mäkitie O . Recent discoveries in monogenic disorders of childhood bone fragility. Curr Osteoporos Rep. 2017;15:303‐310. 28646443 10.1007/s11914-017-0388-6

[2] Mortier GR , Cohn DH , Cormier‐Daire V , et al. Nosology and classification of genetic skeletal disorders: 2019 revision. Am J Med Genet A. 2019;179:2393‐2419. 31633310 10.1002/ajmg.a.61366

[3] Marini JC , Blissett AR . New genes in bone development: what's new in osteogenesis imperfecta. J Clin Endocrinol Metab. 2013;98:3095‐3103. 23771926 10.1210/jc.2013-1505 PMC3733862

[4] Rivadeneira F , Mäkitie O . Osteoporosis and bone mass disorders: from gene pathways to treatments. Trends Endocrinol Metab. 2016;27:262‐281. 27079517 10.1016/j.tem.2016.03.006

[5] Laine CM , Joeng KS , Campeau PM , et al. WNT1 mutations in early‐onset osteoporosis and osteogenesis imperfecta. N Engl J Med. 2013;368:1809‐1816. 23656646 10.1056/NEJMoa1215458 PMC3709450

[6] van Dijk FS , Zillikens MC , Micha D , et al. PLS3 mutations in X‐linked osteoporosis with fractures. N Engl J Med. 2013;16:1529‐1536. 10.1056/NEJMoa1308223 24088043

[7] Laine CM , Wessman M , Toiviainen‐Salo S , et al. A novel splice mutation in PLS3 causes X‐linked early onset low‐turnover osteoporosis. J Bone Miner Res. 2015;3:510‐518. 10.1002/jbmr.2355 25209159

[8] Munns CF , Fahiminiya S , Poudel N , et al. Homozygosity for frameshift mutations in XYLT2 result in a spondylo‐ocular syndrome with bone fragility, cataracts, and hearing defects. Am J Hum Genet. 2015;6:971‐978. 10.1016/j.ajhg.2015.04.017 PMC4457947 26027496

[9] Estrada K , Styrkarsdottir U , Evangelou E , et al. Genome‐wide meta‐analysis identifies 56 bone mineral density loci and reveals 14 loci associated with risk of fracture. Nat Genet. 2012;5:491‐501. 10.1038/ng.2249 PMC3338864 22504420

[10] Hsu YH , Kiel DP . Clinical review: genome‐wide association studies of skeletal phenotypes: what we have learned and where we are headed. J Clin Endocrinol Metab. 2012;10:E1958‐E1977. 10.1210/jc.2012-1890 PMC3674343 22965941

[11] Morris JA , Kemp JP , Youlten SE , et al. An atlas of genetic influences on osteoporosis in humans and mice. Nat Genet. 2019;51:258‐266. 30598549 10.1038/s41588-018-0302-x PMC6358485

[12] Kemp JP , Morris JA , Medina‐Gomez C , et al. Identification of 153 new loci associated with heel bone mineral density and functional involvement of GPC6 in osteoporosis. Nat Genet. 2017;10:1468‐1475. 10.1038/ng.3949 PMC5621629 28869591

[13] Mäkitie RE , Kämpe A , Costantini A , Alm JJ , Magnusson P , Mäkitie O . Biomarkers in WNT1 and PLS3 osteoporosis: altered concentrations of DKK1 and FGF23. J Bone Miner Res. 2020;35:901‐912. 31968132 10.1002/jbmr.3959

[14] Genant HK , Wu CY , van Kuijk C , Nevitt MC . Vertebral fracture assessment using a semiquantitative technique. J Bone Miner Res. 1993;8:1137‐1148. 8237484 10.1002/jbmr.5650080915

[15] Mäyränpää MK , Tamminen IS , Krӧger H , Mäkitie O . Bone biopsy findings and correlation with clinical, radiological, and biochemical parameters in children with fractures. J Bone Miner Res. 2011;26:1748‐1758. 21351145 10.1002/jbmr.373

[16] Dempster DW , Compston JE , Drezner MK , et al. Standardized nomenclature, symbols, and units for bone histomorphometry: a 2012 update of the report of the ASBMR Histomorphometry Nomenclature Committee. J Bone Miner Res. 2013;1:2‐17. 10.1002/jbmr.1805 PMC3672237 23197339

[17] Recker RR , Kimmel DB , Parfitt AM , Davies KM , Keshawarz N , Hinders S . Static and tetracycline‐based bone histomorphometric data from 34 normal postmenopausal females. J Bone Miner Res. 1988;2:133‐144. 10.1002/jbmr.5650030203 3213608

[18] Paila U , Chapman BA , Kirchner R , Quinlan AR . GEMINI: integrative exploration of genetic variation and genome annotations. PLoS Comput Biol. 2013;9:e1003153. 23874191 10.1371/journal.pcbi.1003153 PMC3715403

[19] Forlino A , Marini JC . Osteogenesis imperfecta. Lancet. 2016;387:1657‐1671. 26542481 10.1016/S0140-6736(15)00728-X PMC7384887

[20] Mäkitie RE , Costantini A , Kämpe A , Alm JJ , Mäkitie O . New insights into monogenic causes of osteoporosis. Front Endocrinol (Lausanne). 2017;10:70. 10.3389/fendo.2019.00070 PMC6397842 30858824

[21] 1000 Genomes Project Consortium , Auton A , Brooks LD , Durbin RM , et al. A global reference for human genetic variation. Nature. 2015;7571:68‐74. 10.1038/nature15393 PMC4750478 26432245

[22] Lek M , Karczewski KJ , Minikel EV , et al. Analysis of protein‐coding genetic variation in 60,706 humans. Nature. 2016;7616:285‐291. 10.1038/nature19057 PMC5018207 27535533

[23] SIFT missense predictions for genomes . Nat Protocols. 2016;11:1‐9. 10.1038/nprot.2015.123 26633127

[24] Schwarz JM , Cooper DN , Schuelke M , Seelow D . MutationTaster2: mutation prediction for the deep‐sequencing age. Nat Methods. 2014;11:361‐362. 24681721 10.1038/nmeth.2890

[25] Ioannidis NM , Rothstein JH , Pejaver V , et al. REVEL: an ensemble method for predicting the pathogenicity of rare missense variants. Am J Hum Genet. 2016;4:877‐885. 10.1016/j.ajhg.2016.08.016 PMC5065685 27666373

[26] Costantini A , Skarp S , Kämpe A , et al. Rare copy number variants in array‐based comparative genomic hybridization in early‐onset skeletal fragility. Front Endocrinol. 2018;9:380. 10.3389/fendo.2018.00380 PMC6048219 30042735

[27] Trajanoska K , Morris JA , Oei L , et al. Assessment of the genetic and clinical determinants of fracture risk: genome wide association and mendelian randomisation study. BMJ. 2018;362:k3225. 30158200 10.1136/bmj.k3225 PMC6113773

[28] Medina‐Gomez C , Kemp JP , Trajanoska K , et al. Life‐course genome‐wide association study meta‐analysis of total body BMD and assessment of age‐specific effects. Am J Hum Genet. 2018;102:88‐102. 29304378 10.1016/j.ajhg.2017.12.005 PMC5777980

[29] Zheng HF , Forgetta V , Hsu YH , et al. Whole‐genome sequencing identifies EN1 as a determinant of bone density and fracture. Nature. 2015;526:112‐117. 26367794 10.1038/nature14878 PMC4755714

[30] Thuault S , Comunale F , Hasna J , et al. The RhoE/ROCK/ARHGAP25 signaling pathway controls cell invasion by inhibition of Rac activity. Mol Biol Cell. 2016;17:2653‐2661. 10.1091/mbc.E16-01-0041 PMC5007086 27413008

[31] Jiu Y , Peränen J , Schaible N , et al. Vimentin intermediate filaments control actin stress fiber assembly through GEF‐H1 and RhoA. J Cell Sci. 2017;130:892‐902. 28096473 10.1242/jcs.196881 PMC5358333

[32] Venselaar H , Te Beek TA , Kuipers RK , Hekkelman ML , Vriend G . Protein structure analysis of mutations causing inheritable diseases. An e‐Science approach with life scientist friendly interfaces. BMC Bioinformatics. 2010;11:548. 21059217 10.1186/1471-2105-11-548 PMC2992548

[33] Christov M , Jüppner H . Phosphate homeostasis disorders. Best Pract Res Clin Endocrinol Metab. 2018;5:685‐706. 10.1016/j.beem.2018.06.004 30449549

[34] Csepanyi‐Kömi R , Sirokmany G , Geiszt M , Ligeti E . ARHGAP25, a novel Rac GTPase‐activating protein, regulates phagocytosis in human neutrophilic granulocytes. Blood. 2012;119:573‐582. 22096251 10.1182/blood-2010-12-324053

[35] Ridley AJ , Hall A . The small GTP‐binding protein rho regulates the assembly of focal adhesions and actin stress fibers in response to growth factors. Cell. 1992;70:389‐399. 1643657 10.1016/0092-8674(92)90163-7

[36] Katoh M , Katoh M . Identification and characterization of ARHGAP24 and ARHGAP25 genes in silico. Int J Mol Med. 2004;14:333‐338. 15254788

[37] Etienne‐Manneville S , Hall A . Rho GTPases in cell biology. Nature. 2002;420:629‐635. 12478284 10.1038/nature01148

[38] Hall A . Rho GTPases and the actin cytoskeleton. Science. 1998;279:509‐514. 9438836 10.1126/science.279.5350.509

[39] Tcherkezian J , Lamarche‐Vane N . Current knowledge of the large RhoGAP family of proteins. Biol Cell. 2007;99:67‐86. 17222083 10.1042/BC20060086

[40] Hanna S , El‐Sibai M . Signaling networks of Rho GTPases in cell motility. Cell Signal. 2013;25:1955‐1961. 23669310 10.1016/j.cellsig.2013.04.009

[41] Huang GH , Sun ZL , Li HJ , Feng DF . Rho GTPase‐activating proteins: Regulators of Rho GTPase activity in neuronal development and CNS diseases. Mol Cell Neurosci. 2017;80:18‐31. 28163190 10.1016/j.mcn.2017.01.007

[42] Moon SY , Zheng Y . Rho GTPase‐activating proteins in cell regulation. Trends Cell Biol. 2003;13:13‐22. 12480336 10.1016/s0962-8924(02)00004-1

[43] Müller PM , Rademacher J , Bagshaw RD , et al. Systems analysis of RhoGEF and RhoGAP regulatory proteins reveals spatially organized RAC1 signalling from integrin adhesions. Nat Cell Biol. 2020;4:498‐511. 10.1038/s41556-020-0488-x 32203420

[44] Sanz‐Moreno V , Gadea G , Ahn J , et al. Rac activation and inactivation control plasticity of tumor cell movement. Cell. 2008;3:510‐523. 10.1016/j.cell.2008.09.043 18984162

[45] Wu X , Tu X , Joeng KS , Hilton MJ , Williams DA , Long F . Rac1 activation controls nuclear localization of beta‐catenin during canonical Wnt signaling. Cell. 2008;2:340‐353. 10.1016/j.cell.2008.01.052 PMC2390926 18423204

[46] Weivoda MM , Oursler MJ . The roles of small GTPases in osteoclast biology. Orthop Muscular Syst. 2014;3:1000161. 25599004 10.4172/2161-0533.1000161 PMC4296324

[47] Touaitahuata H , Blangy A , Vives V . Modulation of osteoclast differentiation and bone resorption by Rho GTPases. Small GTPases. 2014;5:e28119. 24614674 10.4161/sgtp.28119 PMC4114643

[48] Wang Y , Lebowitz D , Sun C , Thang H , Grynpas MD , Glogauer M . Identifying the relative contributions of Rac1 and Rac2 to osteoclastogenesis. J Bone Miner Res. 2008;23:260‐270. 17922611 10.1359/jbmr.071013

[49] Itzstein C , Coxon FP , Rogers MJ . The regulation of osteoclast function and bone resorption by small GTPases. Small GTPases. 2011;2:117‐130. 21776413 10.4161/sgtp.2.3.16453 PMC3136942

[50] Ory S , Brazier H , Pawlak G , Blangy A . Rho GTPases in osteoclasts: orchestrators of podosome arrangement. Eur J Cell Biol. 2008;87:469‐477. 18436334 10.1016/j.ejcb.2008.03.002

[51] Razzouk S , Lieberherr M , Cournot G . Rac‐GTPase, osteoclast cytoskeleton and bone resorption. Eur J Cell Biol. 1999;78:249‐255. 10350213 10.1016/S0171-9335(99)80058-2

[52] Croke M , Ross FP , Korhonen M , Williams DA , Zou W , Teitelbaum SL . Rac deletion in osteoclasts causes severe osteopetrosis. J Cell Sci. 2011;124:3811‐3821. 22114304 10.1242/jcs.086280 PMC3225269

[53] Ito Y , Teitelbaum SL , Zou W , et al. Cdc42 regulates bone modeling and remodeling in mice by modulating RANKL/M‐CSF signaling and osteoclast polarization. J Clin Invest. 2010;6:1981‐1993. 10.1172/JCI39650 PMC2877929 20501942

[54] Itokowa T , Zhu ML , Troiano N , Bian J , Kawano T , Insogna K . Osteoclasts lacking Rac2 have defective chemotaxis and resorptive activity. Calcif Tissue Int. 2010;1:75‐86. 10.1007/s00223-010-9435-3 PMC3155765 21110188

[55] Lane SW , De Vita S , Alexander KA , et al. Rac signaling in osteoblastic cells is required for normal bone development but is dispensable for hematopoietic development. Blood. 2011;3:736‐744. 10.1182/blood-2011-07-368753 PMC3265198 22123845

[56] Onishi M , Fujita Y , Yoshikawa H , Yamashita T . Inhibition of Rac1 promotes BMP‐2‐induced osteoblastic differentiation. Cell Death Dis. 2013;4:e698. 23807227 10.1038/cddis.2013.226 PMC3702300

[57] Huck K , Sens C , Wuerfel C , Zoeller C , Nakchbandi IA . The Rho GTPase RAC1 in osteoblasts controls their function. Int J Mol Sci. 2020;21:385. 10.3390/ijms21020385 PMC7014472 31936261

[58] Wan Q , Cho E , Yokota H , Na S . Rac1 and Cdc42 GTPases regulate shear stress‐driven β‐catenin signaling in osteoblasts. Biochem Biophys Res Commun. 2013;433:502‐507. 23524265 10.1016/j.bbrc.2013.03.020 PMC3648988

[59] Yokota K , Sato K , Miyazaki T , et al. Characterization and function of tumor necrosis factor alpha and interleukin‐6‐induced osteoclasts in rheumatoid arthritis [published online ahead of print January 29, 2021]. Arthritis Rheumatol. 2021. 10.1002/art.41666. PMC8361923 33512089

[60] Basel D , Steiner RD . Osteogenesis imperfecta: recent findings shed new light on this once well‐understood condition. Genet Med. 2009;11:375‐385. 19533842 10.1097/GIM.0b013e3181a1ff7b

[61] Kocijan R , Muschitz C , Fratzl‐Zelman N , et al. Femoral geometric parameters and BMD measurements by DXA in adult patients with different types of osteogenesis imperfecta. Skeletal Radiol. 2013;42:187‐194. 22955449 10.1007/s00256-012-1512-4

[62] Mäkitie RE , Haanpää M , Valta H , et al. Skeletal characteristics of WNT1 osteoporosis in children and young adults. J Bone Miner Res. 2016;31:1734‐1742. 27005318 10.1002/jbmr.2841

[63] Qin L , Liu W , Cao H , Xiao G . Molecular mechanosensors in osteocytes. Bone Res. 2020;8:23. 10.1038/s41413-020-0099-y PMC7280204 32550039

[64] Staines KA , Prideaux M , Allen S , Buttle DJ , Pitsillides AA , Farquharson C . E11/podoplanin protein stabilization through inhibition of the proteasome promotes osteocyte differentiation in murine in vitro models. J Cell Physiol. 2016;231:1392‐1404. 26639105 10.1002/jcp.25282 PMC4832367

[65] Suzuki D , Bush JR , Bryce DM , Kamijo R , Beier F . Rac1 dosage is crucial for normal endochondral bone growth. Endocrinology. 2017;158:3386‐3398. 28977598 10.1210/en.2016-1691

[66] Gossiel F , Finigan J , Jacques R , et al. Establishing reference intervals for bone turnover markers in healthy postmenopausal women in a nonfasting state. Bonekey Rep. 2014;3:573. 25228986 10.1038/bonekey.2014.68 PMC4162466

