# Supplementary material for: An ARHGAP25 variant links aberrant Rac1 function to early‐onset skeletal fragility
Source: JBMR Plus. 2021 Jun 7;5(7):e10509. doi: 10.1002/jbm4.10509 (PMC8260816; doi:10.1002/jbm4.10509)
Supplement: Supplementary file 1 — Appendix S1: Supplementary Materials [file JBM4-5-e10509-s001.docx]

**An *ARHGAP25* variant links aberrant RhoGTPase function to**

**early-onset osteoporosis**

**Supplementary material**

Riikka E. Mäkitie MD PhD^1,2,3^, Petra Henning PhD^4^, Yaming Jiu PhD^5,6,7^, Anders Kämpe MD PhD ^8^, Konstantin Kogan PhD^5^, Alice Costantini MSc PhD^8^, Ville-Valtteri Välimäki MD PhD ^9^, Carolina Medina-Gomez PhD^10^, Minna Pekkinen PhD^1,2^, Isidro B. Salusky MD^11^, Camilla Schalin-Jäntti MD PhD ^12^, Maria K. Haanpää MD PhD ^13^, Fernando Rivadeneira MD PhD^10^, J. H. Duncan Bassett MD PhD ^3^, Graham R. Williams MD PhD ^3^, Ulf H. Lerner DDS PhD^4^, Renata C. Pereira PhD^11^, Pekka Lappalainen MSc PhD^5^, Outi Mäkitie, MD PhD ^1,2,8,14^

^1^ Folkhälsan Institute of Genetics, Helsinki, Finland

^2^ Research Program for Clinical and Molecular Metabolism, Faculty of Medicine, University of Helsinki, Helsinki, Finland

^3^ Molecular Endocrinology Laboratory, Department of Metabolism, Digestion and Reproduction, Hammersmith Campus, Imperial College London, London, United Kingdom

^4^ Centre for Bone and Arthritis Research, Department of internal medicine and clinical nutrition, Institute of Medicine, Sahlgrenska Academy, University of Gothenburg, 405 30 Gothenburg, Sweden

^5^ HiLIFE Institute of Biotechnology, University of Helsinki, Helsinki, Finland

^6^ The Center for Microbes, Development and Health, Key Laboratory of Molecular Virology and Immunology, Institut Pasteur of Shanghai, Chinese Academy of Sciences, Shanghai 200031, China

^7^ University of Chinese Academy of Sciences, Beijing, China

^8^ Department of Molecular Medicine and Surgery and Center for Molecular Medicine, Karolinska Institutet, Stockholm, Sweden

^9^ Department of Orthopaedics and Traumatology, Helsinki University Central Hospital and Helsinki University, Jorvi Hospital, Espoo, Finland

^10^ Department of Internal Medicine, Erasmus MC, University Medical Center Rotterdam, The Netherlands

^11^ Department of Pediatrics, David Geffen School of Medicine at UCLA, Los Angeles, CA

^12^ Endocrinology, Abdominal Center, University of Helsinki and Helsinki University Hospital, Helsinki, Finland

^13^ Department of Genomics and Clinical Genetics, Turku University Hospital, Turku, Finland a

^14^ Children’s Hospital, University and Helsinki University Hospital, Helsinki, Finland

**Address all correspondence and requests for reprints to:**

Riikka E Mäkitie, MD PhD

Folkhälsan Institute of Genetics, P.O. Box 63,

FIN-00014 University of Helsinki, Helsinki, FINLAND

E-mail: riikka.makitie@helsinki.fi

Tel. +358-2941911

**Supplementary material**

**1. Supplementary materials and methods**

1.1 Genetic studies 4

*Exome sequencing* 4

*Linkage analysis* 4

1.2 Protein studies 4

*Cell culture and transfections* 4

*Plasmids* 5

*Rac1 activity assay*  6

*Immunofluorescence microscopy* 6

*Cell spreading assay* 7

*EGF-stimulation* 7

*Structure modelling* 7

1.3 *In vitro* studies on murine bone cells 8

1.4 *In vivo* expression of *Arhgap25* in mouse tissues 9

1.5 *In vitro* studies on human osteoclasts 10

**2. Supplementary figures**

Figure S1. Additional biochemistry 13

Figure S2. *Arhgap25* expression in mouse tissues 14

Figure S3. *Arhgap25* expression in murine bone cells 15

Figure S4. *ARHGAP25* expression in osteoclasts 16

Figure S5. LOD scores from linkage analysis 17

**3. Supplementary tables**

Table S1. Remaining variants after filtering of WES data 18

Table S2. Heterozygous variants remaining after filtering in a 19

Finnish family with autosomal dominant primary osteoporosis

Table S3. Variants found in genes associated with phosphate homeostasis disorders in a 20

Finnish family with autosomal dominant primary osteoporosis

Table S4. Top 12 variants found in *ARHGAP25* for association with heel BMD 21

**4. Supplementary references** 22

**1. Supplementary materials and methods**

1.1 Genetic studies

*Exome sequencing*

Exome sequencing was performed at Oxford Gene Technology (OGT, Oxfordshire, UK) according to their standard methods. Genomic DNA was sequenced using TruSeq v 3 on a HiSeq2000 system (Illumina, San Diego, CA, USA), reads were aligned to human reference genome GRCh37 using Burrows-Wheeler Aligner (BWA) (1), duplicates were marked with Picard (v1.107) (http://broadinstitutegithubio/picard), base quality scores were recalibrated using the GATK-sutie (2), and variants were called with FreeBayes (3). Vcflib (4) and vt (5) were used for variant decomposition and left normalization. Variant annotation was performed using Variant Effect Predictor (version 84) (6) and variant exploration using the GEMINI framework (0.19.0) (7).

*Linkage analysis*

To statistically quantify the association with the candidate variant in *ARHGAP25* with the family's skeletal phenotype, we performed a linkage analysis and LOD score calculation. For the analysis, mutation-positive adults were labelled as "affected", mutation-negative adults (including non-related family members) as "unaffected", and four children (IV-1–IV-4) as "phenotype unknown". With the two female adults (subjects III-5 and III-6), we calculated two scenarios in regard to their milder phenotype: for scenario 1 both were labelled as "unaffected" and for scenario 2 as "phenotype unknown". Further, calculations were performed for both 100% penetrance and 90% penetrance of the variant.

1.2 Protein studies

*Cell culture and transfections*

Human osteosarcoma (U2OS) cells were maintained as described in Jiu *et al* (8). Briefly, cells were maintained at +37°C in a humidified atmosphere with 5% CO2 in Dulbecco's modified Eagle's medium (DMEM) (BE12-614F, Lonza, Basel, Switzerland) supplemented with 10% fetal bovine serum (10500-064, Gibco), 10 U/ml Penicillin, 10 µg/ml streptomycin and 20 mM L-Glutamine (from 100X concentrate, Gibco). Starvations were done in the same cell culture medium without fetal bovine serum. Transient transfections were performed with Fugene HD (Promega, Madison, WI, USA) according to manufacturer’s instructions using 3.5:1 Fugene to DNA ratio and 24-hour incubation prior fixation with 4% paraformaldehyde (PFA) in phosphate buffered saline (PBS) for imaging.

*Plasmids*

To determine the effect of the *ARHGAP25* mutation on protein function, we compared changes in actin organization, cellular spreading, and Rac1 activity between WT ARHGAP25, a known inactive form (ARHGAP25 R193A) and our mutant protein. Constructs for GFP-ARHGAP25 R193A, GFP-ARHGAP25 WT and control vector pEGFP-C3 were kind gifts from Sylvie Thuault and Cécile Gauthier-Rouvière (Université de Montpellier, Montpellier, France). These constructs were all generated originally in the ARHGAP25 isoform C and, thus, we also generated the Finnish family mutation *ARHGAP25* c.631G>A (p.Gly211Arg) in isoform C (639aa, NM_001166276.1, NP_001159748.1). Isoform C only differs from the canonical ARHGAP25 isoform A in the 5’UTR and proximal coding region (9): amino acids 4–19 in isoform A are replaced by differing amino acids 4–12 in isoform C and shown in blue; the 5’UTR is shown in yellow; conserved coding region in purple. The difference in N-terminal sequence in isoforms A and C do not affect any functional domains described within ARHGAP25.

**Isoform A (NM_001007231.3)**

301 CAAGAAGGGNNCAAACNGTGANNGANTCANNNCTTCANTNACTANTCNCTTANACNGNAN 360

............................................................

............................................................

361 GNAAANTGTCNNTAAAATTNCNAAGNAACTNGNNTNTCNNCCTGAAANTGGAGGCTGNNA 420

.....ATGTCCCTAAAATTGCCAAGGAACTGGGATTTCAACCTGAAAGTGGAGGCTGCGA 55

.....‑M‑‑S‑‑L‑‑K‑‑L‑‑P‑‑R‑‑N‑‑W‑‑D‑‑F‑‑N‑‑L‑‑K‑‑V‑‑E‑‑A‑‑A‑‑ 18

421 AAATAGCTCGNTCAAGGANTGTGNTGACNGGNNAGCANATNGCTNCCTTCCATCNNTNNT 480

56 AAATAGCTCGGTCAAGGAGTGTGATGACTGGCGAGCAGATGGCTGCCTTCCATCCATCGT 115

19 K‑‑I‑‑A‑‑R‑‑S‑‑R‑‑S‑‑V‑‑M‑‑T‑‑G‑‑E‑‑Q‑‑M‑‑A‑‑A‑‑F‑‑H‑‑P‑‑S‑‑ 38

**Isoform C (NM_001166276.1)**

301 GACCTNGTTGTCNTCNCNCCGCTTCTCAGNCCCCNCTGGGNTTCCTCTNTNCNCTNCNCC 360

............................................................

............................................................

361 NACTNNTTNNTCANTGNCCCNTGNNCCTNNGTCANTCNNNNTGNCTGTTCCTCTNTATAG 419

....................ATGTCCCTCGGTCAGTCGGCCTGTCTGTTCCTCTCTATAG 40

....................‑M‑‑S‑‑L‑‑G‑‑Q‑‑S‑‑A‑‑C‑‑L‑‑F‑‑L‑‑S‑‑I‑‑ 13

420 CTCGNTCAAGGANTGTGNTGACNGGNNAGCANATNGCTNCCTTCCATCNNTNNTCCNNNN 479

41 CTCGGTCAAGGAGTGTGATGACTGGCGAGCAGATGGCTGCCTTCCATCCATCGTCCACCC 100

14 A‑‑R‑‑S‑‑R‑‑S‑‑V‑‑M‑‑T‑‑G‑‑E‑‑Q‑‑M‑‑A‑‑A‑‑F‑‑H‑‑P‑‑S‑‑S‑‑T‑‑ 33

The GFP-ARHGAP25 G211R mutagenesis (G631A in nucleotide sequence and corresponding to the G218R in patients) was introduced based on GFP-ARHGAP25 WT. We used PCR (KAPA HiFi ReadyMix system, Roche, Madison, WI, USA) to amplify the template GFP-ARHGAP25 WT. PCR primers were: Forward primer 5’–CGCTTTTGATGCTAGGGAGCGGCCCTCCTTTGACAG–3’ and reverse primer 5’–CCGCTCCCTAGCATCAAAAGCGTCTCTCAGCTGCTTC–3’. The PCR product was further incubated with FD-Dpn1 (ER1705, Thermo Scientific, Waltham, MA, USA) to destroy the original template.

*Rac1 activity assay*

Rac1 activity was measured using a Rac1 G-LISA absorbance-based biochemical assay kit (Cytoskeleton, Denver, CO, USA) according to the manufacturer’s instructions. In brief, cells were lysed, aliquoted and snap frozen in liquid nitrogen. After rapid thawing, binding buffer was added to the cell lysate, which was subsequently incubated on a Rac1-GTP affinity plate coated with Rac1-GTP-binding protein in each well. The plate was placed on an orbital plate shaker at 400 rpm for 30 min at 4°C. After washes, primary anti-Rac1 antibodies (1:50) and secondary HRP-linked antibodies (1:100) were sequentially added to the wells followed by an incubation on an orbital shaker at 400 rpm for 45 min at room temperature. Thereafter, the signal was developed with HRP-detection reagents. The absorbance was measured by means of a plate reader spectrophotometer Enspire (PerkinElmer) at 490 nm. In the quantifications, the absorbance values of control vector-expressing cells were set to 1 in each experiment, and the differences between the control and the ARHGAP25 wild-type, G211R-(corresponding to the G218R in patients) and R193A-overexpressing cells in corresponding experiments were calculated.

*Immunofluorescence microscopy*

For immunofluorescence, cells were fixed with 4% PFA-PBS for 20 min at room temperature, washed three times with 0.2% Dulbecco/BSA and permeabilized with 0.1% Triton X-100 in TBS. F-actin was visualized with Alexa Fluor 568-phalloidin (dilution 1:200; A1238, Thermo Fisher, Waltham, MA, USA) and nuclei were stained with DAPI (1 µg/ml; Sigma, Espoo, Finland). All immunofluorescence data were obtained with DM 6000B (Leica, Wetzlar, Germany) using 63x/1.40-0.60 HCX PL APO objective, Semrock Brightline filters: DAPI-5060C (ex 377/50, em 447/60), GFP-4050B (ex 466/40, em 525/50), TRITC-B (ex 543/22, em 593/40) and Hamamatsu Orca-Flash4.0 V2 sCMOS camera.

*Cell spreading assay*

After transient transfection, the cells were incubated for 24 hours and re-plated on fibronectin-coated (10 μg/ml) or non-coated glass cover slips. After incubation for 5 hr for non-coated and 1 hr for fibronectin-coated conditions, cells were then fixed for immunofluorescence. Cell outlines were visualized by phalloidin-stained actin and cell areas were calculated using ImageJ. Statistical analyses were performed with SigmaPlot 11.0 (‎Systat Software).

*EGF-stimulation*

After transient transfection, the cells were incubated for 24 hours and stimulation was carried out with 0.1 µg/ml epidermal growth factor (EGF) (E9644, Lot#065M4070V, Sigma, Espoo, Finland) for 30 minutes. Cells were then immediately fixed for immunofluorescence or harvested for G-LISA assay.

*Structure modelling*

Homologous modelling of ARHGAP25 was performed using Phyre2 server. RHOGAP1 (PDBID 1TX4) was used as a template to model ARHGAP25. The combined structural model of ARHGAP25 is composed of a PH domain, a short flexible linker and a GAP domain. The PH domain was obtained from a previously described NMR model (PDBID: 1V89). The linker was modelled based on the threading of the original ARHGAP25 sequence onto existing extensions taken from the 1V89 NMR model. Homologue modelling of the GAP domain of ARHGAP25 was performed on Phyre2 server using the RHOGAP1 (PDBID 1TX4) chain A structure as a template since it has the highest sequence homology. The GTPase was positioned based on superimposition of the RHOGAP1-RHO complex (PDBID 1TX4) to the modelled GAP domain of ARHGAP25.

1.3 *In vitro* studies on murine bone cells

*Arhgap25* gene expression was also analyzed in cultured mouse osteoclasts and osteoblasts. For osteoclast differentiation studies, mouse bone marrow cells were flushed from the tibia and femur of 8–12-week-old *C57BL/6* mice. Bone macrophages were isolated and expanded as previously described (10). Briefly, bone marrow cells were cultured in suspension culture dishes (Corning, 430591) in complete α-MEM in the presence of 30 ng/ml murine M-CSF (cat no 416-ML-050, R&D Systems, Minneapolis, MN, USA) for 2 days. Subsequently, the adherent macrophages were detached, and seeded at 5.000 cells/96 well and cultured in complete α-MEM in the presence of 30 ng/ml murine M-CSF with or without 4 ng/ml recombinant mouse RANKL for 72 h. Murine calvarial osteoblasts were isolated by sequential collagenase treatment of calvarias dissected from 3–5 days old *C57BL/6* mice as previously described (11,12). Fractions 5–10 were used and expanded for 3 days in complete α-MEM. Thereafter, the cells were detached and seeded at a density of 20.000 cells/cm^2^ in 48-well plates and cultured in osteogenic media (complete α-MEM supplemented with 2 mM β-glycerophosphate and 0.2 mM L-Ascorbic acid 2-phosphate sesquimagnesium salt) for 2 or 7 days. Murine bone cells were harvested and RNA purification, cDNA synthesis and gene expression analysis were performed as for human osteoclasts. Gene expression was analyzed using predesigned TaqMan Assays (*Arhgap25*: Mm00615449_m1, *Acp5*: Mm00475698_m1, *Alpl*: Mm00475834_m1) with 18S rRNA as internal control. The mouse *Arhgap25* assay covers the exon 6–7 boundary in RefSeq NM_001037727.2, which is identical in both *Arhgap25* isoforms A and C. Comparable expression of the 18S gene used as internal standard was verified by calculating the relative expression of 18S versus ng RNA converted to cDNA used in the TaqMan Assays (Figure S3F).

1.4 *In vivo* expression in of *Arhgap25* in mouse tissues

The mRNA expression of *Arhgap25* in different tissues was evaluated in 22-week-old male *C57BL/6* mice (Charles River). Mice where anesthetized with Ketador/Dexdomitor (Richter Pharma, Wels, Austria/Orion Pharma, Espoo Finland), bled, and euthanized by cervical dislocation. Soft tissues were dissected, snap-frozen in liquid nitrogen, and stored at -80C until RNA preparation. Flushed mid-diaphyseal tibial bone (cortical bone) and vertebral body L6 were stored in RNAlater at -80°C until RNA preparation. Liver, kidney, spleen, lung, muscle, heart, aorta, thymus, testes and seminal vesicle were homogenized in RLT buffer with 1% 2-mercaptoethanol using a TissueLyser, followed by RNA preparation using the RNeasy mini kit (Qiagen, Carlsbad, CA, USA). Bone tissues (cortical bone and vertebra), fat (retroperitoneal, gonadal and brow fat), bone marrow, brain cortex and hypothalamus were homogenized in TRIzol reagent (Life Technologies, Carlsbad, CA, USA) using a Tissue Lyser. After centrifugation to remove cell debris, the TRIzol homogenate was mixed with an equal volume of chloroform, centrifuged and the aqueous phase recovered. The aqueous phase was mixed with an equal volume of 70% ethanol and added to an RNeasy spin column. Thereafter the isolation followed the protocol from the RNeasy mini Kit (Qiagen, Hilden, Germany). RNA was converted to cDNA using High-Capacity cDNA Reverse Transcription kit (Applied Biosystems, Foster City, CA, USA). Quantitative real-time PCR analysis for *Arhgap25* was performed in the StepOnePlus Real-Time PCR system using pre-designed TaqMan Assay (Assay no Mm00615449_m1) and 18S rRNA as internal control. Data are displayed as fold versus cortical bone.

1.5 *In vitro* studies on human osteoclasts

Peripheral blood mononuclear cells were purified by Ficoll-Paque PLUS (GE Healthcare, Helsinki, Finland) centrifugation and CD14^+^ monocytes were subsequently isolated using CD14 MicroBeads and MACS column separation (Miltenyi Biotec, Lund, Sweden) according to the manufacturers’ instructions. CD14^+^ monocytes were seeded in 96-well plates (3x10^5^ cells/cm^2^) in complete α-MEM medium (α-MEM (cat no 22561-021, Gibco, Waltham, MA, USA) supplemented with 10% heat inactivated fetal bovine serum (FBS, cat no F7524, Sigma, Espoo, Finland), 2 mM GlutaMAX (cat no 35050-038, Gibco, Waltham, MA, USA), 50 µg/ml gentamicin (cat no 15750-037, Gibco, Waltham, MA, USA), 100 U/ml penicillin and 100 µg/ml streptomycin (cat no 15140-148, Gibco, Waltham, MA, USA) in the presence of 30 ng/ml human M-CSF (M, cat no 216-MC-025/CF, R&D Systems, Minneapolis, MN, USA) and 2 ng/ml recombinant mouse RANKL (RL, cat no 462-TEC-010, R&D Systems) to induce osteoclastogenesis. Osteoclast studies were performed on both plastic and on discs of bovine bone to study resorption (cat no TDT-1BON1000-96, IDS Immunodiagnostics Systems, Boldon, UK). Media were replenished every third day. At indicated time points of culture, cells were fixed and stained for tartrate resistant acid phosphatase (TRAP) using the Acid Phosphatase, Leukocyte (TRAP) Kit from Sigma (cat no 387A). Media were saved during culture for analysis of TRAP5b and C-terminal telopeptides of type I collagen (CTX) using ELISA kits from IDS Immunodiagnostics Systems (cat no SB-TR201A and AC-07F1, respectively). TRAP5b and CTX were analyzed in media collected on day 8, corresponding to the levels released from days 6 to 8. Resorption pits were visualized by reflective light microscopy and toluidine blue staining. Toluidine blue staining for resorption pits on bone discs was performed after removal of cells by sonication in 0.5 M NaOH. Bone discs were stained for 30 s in 1% (w/v) toluidine blue in 1% (w/v) sodium borate solution followed by a brief wash in water. Number of osteoclasts and pit surface per bone disc was counted using the Bioquant OSTEO software connected to a Jenoptic Gryphax camera on a Nikon Eclipse 80i microscope.

Actin ring formation was studied after 8 days of culture on bone discs. Cells were fixed in 4% phosphate buffered formaldehyde, washed with PBS and permeabilized with 0.1% Triton X-100 in PBS. Cells were subsequently stained with rhodamine conjugated phalloidin (cat no R415, Life Technologies, Carlsbad, CA, USA), 5 U/ml in 2% BSA/PBS, for 20 min at 4°C. After washing in PBS, discs were mounted with Prolong Gold Mountant (cat no P-36931, Life Technologies, Carlsbad, CA, USA) and photographs taken using fluorescence microscopy.

Gene expression was analyzed in cells from healthy blood donors cultured as above. After 72 h of culture the cells were lysed in RLT buffer (Qiagen, Hilden, Germany) and total RNA was purified using RNeasy Micro Kit (Qiagen). cDNA synthesis was performed using the High Capacity cDNA Reverse Transcription Kit from Applied Biosystems (Foster City, CA, USA). *ARHGAP25* and *ACP5* gene expression was analyzed using predesigned TaqMan Assays, (Hs01121033_m1 and Hs00356261_m1, Life Technologies) and the StepOnePlus Real-Time PCR system (Applied Biosystems). The human *ARHGAP25* assay covers the exon 5–6 boundary in RefSeq NM_001007231.2, which is identical in both *ARHGAP25* isoforms A and C. Gene expression was adjusted for the expression of 18S ribosomal RNA (Life Technologies 4310893E) used as an internal control.

**2. Supplementary figures**

**Figure S1.** Concentrations of **A)** plasma phosphate (nmol/L), **B)** serum 25OH-vitamin D (nmol/L), and **C)** serum parathyroid hormone (pg/mL) in nine, and **D)** serum intact fibroblast growth factor 23 (pg/mL) and **E)** serum C-terminal fibroblast growth factor 23 (pmol/L) in four mutation-positive subjects with a heterozygous missense mutation p.C218R in *ARHGAP25* compared to corresponding measurements in 35 mutation-negative healthy Finnish subjects. **p*<0.05, ***p*<0.01, Mann–Whitney *U* Test.

**Figure S2.** *Arhgap25* expression in mouse tissues. Gene expression of *Arhgap25* in a panel of tissues from male *C56BL6* mice.

**Figure S3.** *Arhgap25* expression in murine bone cells. Gene expression of **A)** *Arhgap25* and **B)** *Acp5* in cultured murine bone marrow macrophages and osteoclasts. Gene expression of **C)** *Arhgap25* and **D)** *Alpl* in murine calvarial osteoblasts cultured for 48 hours or 7 days in osteogenic media. **E)** Relative expression of *Arhap25* in macrophages, osteoclasts and osteoblasts and **(F)** relative expression of 18S used as an internal control in E. ***p*<0.01, ****p*<0.005, Student’s *t* Test.

**Figure S4.** *ARHGAP25* expression in human osteoclasts. RANKL induced osteoclastogenesis on plastic. Gene expression of **A)** *ACP5* and **B)** *ARHGAP25* in osteoclasts from healthy blood donors cultured on plastic for 24 and 72 hours. (M: M-CSF, M/RL: M-CSF and RANKL). **C)** *Arhgap25* in osteoclasts from mouse bone marrow macrophages cultured on plastic for 72 hours. (M: M-CSF, M/RL: M-CSF and RANKL). **D)** Photographs of TRAP-stained osteoclasts from subjects and healthy controls after 4 days of culture in M-CSF and RANKL on plastic. Scale bars 20 µm. **p*<0.05, ***p*<0.01, Student’s *t* Test.

**Figure S5.** LOD scores from linkage analysis for heterozygous missense *ARHGAP25* variant p.C218G and a skeletal fragility phenotype in a Finnish family. Calculations are performed for two scenarios: two mutation-positive adults with an uncertain phenotype labelled as **A)** unaffected, and **B)** phenotype unknown.

**3. Supplementary tables**

**Table S1.** List of genes underlying osteogenesis imperfecta and primary osteoporosis.

| **Gene symbol** | **Complete name** | **Inheritance** | **Reference** |
| --- | --- | --- | --- |
| *BMP1* | Bone morphogenetic protein I | AR | 13 |
| *COL1A1* | Alpha-1 chain collagen type I | AD | 14 |
| *COL1A2* | Alpha-1 chain collagen type II | AD | 14 |
| *CREB3L1* | cAMP responsive element binding protein 3-like 1 | AR | 15 |
| *CRTAP* | Cartilage-associated protein | AR | 16 |
| *FKBP10* | FK506-binding protein 10 | AR | 17 |
| *IFITM5* | Interferon-induced transmembrane protein 5 | AD | 18, 19 |
| *LEPRE1* | Prolyl 3-hydroxylase 1 | AR | 20 |
| *LRP5* | LDL-receptor related protein 5 | AD/AR | 21 |
| *MBTPS2* | Membrane bound transcription factor peptidase, site 2 | XR | 22 |
| *PLOD2* | Procollagen-lysine, 2-oxoglutarate 6 dioxygenase 2 | AR | 23 |
| *PLS3* | Plastin 3 | XR | 24 |
| *PPIB* | Peptidyl-prolyl isomerase B | AR | 25 |
| *SEC24D* | SEC24-related gene family, member D | AR | 26 |
| *SERPINF1* | Serpin peptidase inhibitor, Clade F, member 1 | AR | 27 |
| *SERPINH1* | Serpin peptidase inhibitor, Clade H, member 1 | AR | 28 |
| *SGMS2* | Sphingomyelin synthase 2 | AD | 29 |
| *SP7* | Sp7 transcription factor | AR | 30 |
| *SPARC* | Secreted protein acidic and cysteine rich | AR | 31 |
| *TMEM38B* | Transmembrane protein 38B | AR | 32 |
| *WNT1* | Wingless-type MMTV integration site family, member 1 | AD/AR | 33 |
| *XYLT2* | Xylosyltransferase 2 | AR | 34 |

AD = autosomal dominant, AR = autosomal recessive.

**Table S2.** Heterozygous variants remaining after filtering in a Finnish family with autosomal dominant skeletal fragility.

|  | **Chr** | **Position^a^** | **Gene** | **Nucleotide**  **change** | **Mutation** | **Mutation type** | **gnomAD aaf all, max** | **gnomAD aaf Finnish** | **Variant** | **CADD** | **PolyPhen** | **SIFT** |
| --- | --- | --- | --- | --- | --- | --- | --- | --- | --- | --- | --- | --- |
| 1 | 2 | 69034590 | *ARHGAP25* | G>A | p.Gly218Arg | MV | absent | absent | None | 27.7 | Probably damaging | Deleterious |
| 2^b^ | 6 | 33423200 | *ZBTB9* | T>C | p.Leu108Pro | MV | 0.0000, 0.0003 | 0.0000 | rs200550865 | 25.3 | Probably damaging | Deleterious |
| 3^c^ | 8 | 36691176 | *KCNU1* | C>T | p.Ala404Val | MV | 0.0005, 0.0019 | 0.0007 | rs189044708 | 18.1 | Benign | Tolerated |
| 4^c^ | 9 | 78790187 | *PCSK5* | G>C | p.Trp681Ser | MV | absent | absent | rs10118321 | 7.9 | Unknown | None |
| 5^c^ | 10 | 21178852 | *NEBL* | C>G | p.Lys60Asn | MV | 0.0040, 0.0100 | 0.0004 | rs41277374 | 25.5 | Probably damaging | Deleterious |
| 6^c^ | 11 | 34477598 | *CAT* | C>T | p.Ala251Val | MV | 0.0000, 0.0001 | 0.0001 | None | 16.1 | Benign | Tolerated |
| 7^c^ | 20 | 3026350 | *GNRH2* | AGCCCC>  AGCCCCGCCC | frameshift | FS | absent | absent | rs67749149 | None | None | None |

^a^ Positions according to GRCh37

^b^ Variant omitted; variant not true when Sanger sequenced

^c^ Variants omitted; associate with a specific disease/phenotype different to that of our subjects’

Chr = chromosome, aaf = allele frequency, MV = missense variant, SRV = splice region variant, FS = frameshift

**Table S3.** Variants found in genes associated with phosphate homeostasis disorders (35) in a Finnish family with autosomal dominant skeletal fragility.

| **Number** | **Chr** | **Position^a^** | **Gene** | **Nucleotide**  **change** | **Variant location** | **Variant type** | **Segregation with phenotype** |
| --- | --- | --- | --- | --- | --- | --- | --- |
| 1 | 6 | 132213059 | *ENPP1* | T>C | 3’UTR | SNV | No |
| 2 | 6 | 48236231 | *VDR* | C>A | 3’UTR | SNV | No |
| 3 | 8 | 48237736 | *VDR* | CA>C | 3’UTR | Indel | No |
| 4 | 9 | 48295083 | *VDR* | C>T | Intron | SNV | No |
| 5 | 10 | 21178852 | *PHEX* | GTT>G | Intron | Indel | No |
| 6 | 11 | 34477598 | *PHEX* | T>C | Intron | SNV | No |

^a^ Positions according to GRCh37

Chr = chromosome, aaf = allele frequency, SNV = single nucleotide polymorphism, Indel = insertion/deletion

**Table S4.** Top 12 variants found in *ARHGAP25* for association with eBMD derived from heel ultrasound (36).

| **Variant** | **Chromosome** | **Base pair** | ***p*-value** | **Allele 1** | **Allele 2** |
| --- | --- | --- | --- | --- | --- |
| rs10048745 | 2 | 68962137 | 4.80 x 10^-12^ | G | A |
| rs10196674 | 2 | 68997153 | 4.90 x 10^-12^ | A | G |
| rs10173408 | 2 | 68997250 | 6.60 x 10^-12^ | G | C |
| rs2280246 | 2 | 68979141 | 2.10 x 10^-11^ | A | C |
| rs2280244 | 2 | 68979302 | 3.30 x 10^-11^ | G | A |
| rs2280243 | 2 | 68979331 | 3.30 x 10^-11^ | G | T |
| rs13426364 | 2 | 68977254 | 3.80 x 10^-11^ | C | T |
| rs11695006 | 2 | 69006495 | 9.30 x 10^-09^ | A | G |
| rs11685672 | 2 | 69002281 | 1.20 x 10^-08^ | C | G |
| rs10208669 | 2 | 69018787 | 3.10 x 10^-08^ | G | A |
| rs12477343 | 2 | 69017904 | 4.70 x 10^-08^ | G | T |
| rs12478131 | 2 | 69018157 | 4.80 x 10^-08^ | C | T |

**4. Supplementary references**

1. Li, H., and Durbin, R. (2010). Fast and accurate long-read alignment with Burrows-Wheeler transform. Bioinformatics (Oxford, England), 5, 589–595.

2. DePristo, M. A., Banks, E., Poplin, R., Garimella, K. V., Maguire, J. R., Hartl, C., Philippakis, A. A., del Angel, G., Rivas, M. A., Hanna, M., et al. (2011). A framework for variation discovery and genotyping using next-generation DNA sequencing data. Nature genetics, 5, 491–498.

3. Garrison, E. Haplotype-based variant detection from short-read sequencing. arXiv:12073907v2. 2012.

4. Garrison, E. Vcflib, a simple C++ library for parsing and manipulating VCF files. https://githubcom/vcflib/vcflib. 2016.

5. Tan, A., Abecasis, G. R., and Kang, H. M. (2015). Unified representation of genetic variants. Bioinformatics (Oxford, England), 13, 2202–2204.

6. McLaren, W., Gil, L., Hunt, S. E., Riat, H. S., Ritchie, G. R., Thormann, A., Flicek, P., and Cunningham, F. (2016). The Ensembl Variant Effect Predictor. Genome biol., 1, 122.

7. Paila, U., Chapman, B. A., Kirchner, R., and Quinlan, A. R. (2013). GEMINI: integrative exploration of genetic variation and genome annotations. PLoS computational biology, 7, e1003153.

8. Jiu, Y., Lehtimaki, J., Tojkander, S., Cheng, F., Jäälinoja, H., Liu, X., Varjosalo, M., Eriksson, J.E., and Lappalainen, P. Bidirectional Interplay between Vimentin Intermediate Filaments and Contractile Actin Stress Fibers. Cell Rep. 2015;11:1511–1518.

9. Yates, A. D., Achuthan, P., Akanni, W., Allen, J., Allen, J., Alvarez-Jarreta, J., Amode, M. R., Armean, I. M., Azov, A. G., Bennett, R., et al. (2020). Ensembl 2020. Nucleic acids research, 48, D682–D688.

10. Takeshita, S., Kaji, K., and Kudo, A. (2000). Identification and characterization of the new osteoclast progenitor with macrophage phenotypes being able to differentiate into mature osteoclasts. Journal of bone and mineral research: the official journal of the American Society for Bone and Mineral Research, 8, 1477–1488.

11. Granholm, S., Henning, P., Lindholm, C., and Lerner, U. H. (2013). Osteoclast progenitor cells present in significant amounts in mouse calvarial osteoblast isolations and osteoclastogenesis increased by BMP-2. Bone, 52, 83–92.

12. [Bakker, A. D](https://www.ncbi.nlm.nih.gov/pubmed/?term=Bakker%20AD%5BAuthor%5D&cauthor=true&cauthor_uid=22130919)., and [Klein-Nulend, J](https://www.ncbi.nlm.nih.gov/pubmed/?term=Klein-Nulend%20J%5BAuthor%5D&cauthor=true&cauthor_uid=22130919). (2012). Osteoblast isolation from murine calvaria and long bones. [Methods](https://www.ncbi.nlm.nih.gov/pubmed/22130919) Mol. Biol., 816, 19-29.

13. Martínez‐Glez, V., Valencia, M., Caparrós‐Martín, J.A., Aglan, M., Temtamy, S., Tenorio, J., Pulido, V., Lindert, U., Rohrbach, M., Eyre, D., et al. (2012), Identification of a mutation causing deficient BMP1/mTLD proteolytic activity in autosomal recessive osteogenesis imperfecta. Hum. Mutat., 33, 343–350.

14. Sillence, D. O., Senn, A., and Danks, D. M. (1979). Genetic heterogeneity in osteogenesis imperfecta. J. Med. Genet., 6, 101-16.

15. Symoens, S., Malfait, F., D'hondt, S., Callewaert, B., Dheedene, A., Steyaert, W., Bächinger, H. P., De Paepe, A., Kayserili, H., and Coucke, P. J. (2013). Deficiency for the ER-stress transducer OASIS causes severe recessive osteogenesis imperfecta in humans. Orphanet J. Rare Dis., 8, 154.

16. Barnes, A. M., Chang, W., Morello, R., Cabral, W. A., Weis, M., Eyre, D. R., Leikin, S., Makareeva, E., Kuznetsova, N., Uveges, T. E., et al. (2006). Deficiency of cartilage-associated protein in recessive lethal osteogenesis imperfecta. N. Eng. J. Med., 26, 2757–2764.

17. Alanay, Y., Avaygan, H., Camacho, N., Utine, G. E., Boduroglu, K., Aktas, D., Alikasifoglu, M., Tuncbilek, E., Orhan, D., Bakar, F. T., et al. (2010). Mutations in the gene encoding the RER protein FKBP65 cause autosomal-recessive osteogenesis imperfecta. Am. J. Hum. Genet., 4, 551–559.

18. Semler, O., Garbes, L., Keupp, K., Swan, D., Zimmermann, K., Becker, J., Iden, S., Wirth, B., Eysel, P., Koerber, F., Schoenau, E., Bohlander, S. K., Wollnik, B., & Netzer, C. (2012). A mutation in the 5'-UTR of IFITM5 creates an in-frame start codon and causes autosomal-dominant osteogenesis imperfecta type V with hyperplastic callus. Am. J. Hum. Genet., 2, 349–357.

19. Cho, T. J., Lee, K. E., Lee, S. K., Song, S. J., Kim, K. J., Jeon, D., Lee, G., Kim, H. N., Lee, H. R., Eom, H. H., et al. (2012). A single recurrent mutation in the 5'-UTR of IFITM5 causes osteogenesis imperfecta type V. Am. J. Hum. Gen., 2, 343–348.

20. Cabral, W. A., Chang, W., Barnes, A. M., Weis, M., Scott, M. A., Leikin, S., Makareeva, E., Kuznetsova, N. V., Rosenbaum, K. N., Tifft, C. J., et al. (2007). Prolyl 3-hydroxylase 1 deficiency causes a recessive metabolic bone disorder resembling lethal/severe osteogenesis imperfecta. Nature genetics, 3, 359–365.

21. Gong, Y., Slee, R. B., Fukai, N., Rawadi, G., Roman-Roman, S., Reginato, A. M., Wang, H., Cundy, T., Glorieux, F. H., Lev, D., et al. (2001). LDL receptor-related protein 5 (LRP5) affects bone accrual and eye development. Cell, 4, 513–523.

22. Lindert, U., Cabral, W. A., Ausavarat, S., Tongkobpetch, S., Ludin, K., Barnes, A. M., Yeetong, P., Weis, M., Krabichler, B., Srichomthong, C., et al. (2016). MBTPS2 mutations cause defective regulated intramembrane proteolysis in X-linked osteogenesis imperfecta. Nat. Commun. 6, 7, 11920.

23. Puig-Hervás, M. T., Temtamy, S., Aglan, M., Valencia, M., Martínez-Glez, V., Ballesta-Martínez, M. J., López-González, V., Ashour, A. M., Amr, K., Pulido, V., et al. (2012). Mutations in PLOD2 cause autosomal-recessive connective tissue disorders within the Bruck syndrome--osteogenesis imperfecta phenotypic spectrum. Hum. Mut., 10, 1444–1449.

24. van Dijk, F. S., Zillikens, M. C., Micha, D., Riessland, M., Marcelis, C. L., de Die-Smulders, C. E., Milbradt, J., Franken, A. A., Harsevoort, A. J., Lichtenbelt, K. D., et al. (2013). PLS3 mutations in X-linked osteoporosis with fractures. N. Engl. J. Med., 16, 1529–1536.

25. Barnes, A. M., Carter, E. M., Cabral, W. A., Weis, M., Chang, W., Makareeva, E., Leikin, S., Rotimi, C. N., Eyre, D. R., Raggio, C. L., and Marini J. C. Lack of cyclophilin B in osteogenesis imperfecta with normal collagen folding. N. Engl. J. Med., 362, 521–8.

26. Garbes, L., Kim, K., Rieß, A., Hoyer-Kuhn, H., Beleggia, F., Bevot, A., Kim, M. J., Huh, Y. H., Kweon, H. S., Savarirayan, R., et al. (2015). Mutations in SEC24D, encoding a component of the COPII machinery, cause a syndromic form of osteogenesis imperfecta. Am. J. Hum. Gen., 3, 432–439.

27. Homan, E. P., Rauch, F., Grafe, I., Lietman, C., Doll, J. A., Dawson, B., Bertin, T., Napierala, D., Morello, R., Gibbs, R., et al. (2011). Mutations in SERPINF1 cause osteogenesis imperfecta type VI. J. Bone Min. Res., 12, 2798–2803.

28. Christiansen, H. E., Schwarze, U., Pyott, S. M., AlSwaid, A., Al Balwi, M., Alrasheed, S., Pepin, M. G., Weis, M. A., Eyre, D. R., and Byers, P. H. (2010). Homozygosity for a missense mutation in SERPINH1, which encodes the collagen chaperone protein HSP47, results in severe recessive osteogenesis imperfecta. Am. J. Hum. Gen., 3, 389–398.

29. Pekkinen, M., Terhal, P. A., Botto, L. D., Henning, P., Mäkitie, R. E., Roschger, P., Jain, A., Kol, M., Kjellberg, M. A., Paschalis, E. P., et al. (2019). Osteoporosis and skeletal dysplasia caused by pathogenic variants in SGMS2. JCI Insight, 4, e126180.

30. Lapunzina, P., Aglan, M., Temtamy, S., Caparrós-Martín, J. A., Valencia, M., Letón, R., Martínez-Glez, V., Elhossini, R., Amr, K., Vilaboa, N., and Ruiz-Perez, V. L. (2010). Identification of a frameshift mutation in Osterix in a patient with recessive osteogenesis imperfecta. Am. J. Hum. Gen., 1, 110–114.

31. Mendoza-Londono, R., Fahiminiya, S., Majewski, J., Care4Rare Canada Consortium, Tétreault, M., Nadaf, J., Kannu, P., Sochett, E., Howard, A., Stimec, J., et al. (2015). Recessive osteogenesis imperfecta caused by missense mutations in SPARC. Am. J. Hum. Gen., 6, 979–985.

32. Shaheen, R., Alazami, A. M., Alshammari, M. J., Faqeih, E., Alhashmi, N., Mousa, N., Alsinani, A., Ansari, S., Alzahrani, F., Al-Owain, M., Alzayed, Z. S., Alkuraya, F. S. (2012). Study of autosomal recessive osteogenesis imperfecta in Arabia reveals a novel locus defined by TMEM38B mutation. J Med Genet., 49, 630–5.

33. Laine, C. M., Joeng, K. S., Campeau, P. M., Kiviranta, R., Tarkkonen, K., Grover, M., Lu, J. T., Pekkinen, M., Wessman, M., Heino, T. J., et al. (2013). WNT1 mutations in early-onset osteoporosis and osteogenesis imperfecta. N. Engl. J. Med., 19, 1809–1816.

34. Munns, C. F., Fahiminiya, S., Poudel, N., Munteanu, M. C., Majewski, J., Sillence, D. O., Metcalf, J. P., Biggin, A., Glorieux, F., Fassier, F., et al. (2015). Homozygosity for frameshift mutations in XYLT2 result in a spondylo-ocular syndrome with bone fragility, cataracts, and hearing defects. Am J Hum Genet., 96, 971­–8.

35. Christov, M., and Jüppner, H. (2018). Phosphate homeostasis disorders. Best Pract. Res. Clin. Endocrinol. Metab. 5, 685–706.

36. Morris, J. A., Kemp, J. P., Youlten, S. E., Laurent, L., Logan, J. G., Chai, R. C., Vulpescu, N. A., Forgetta, V., Kleinman, A., Mohanty, S. T., et al. (2019). An atlas of genetic influences on osteoporosis in humans and mice. Nat. Genet. 51, 258–266.
